# Mechanistic and applied insights into antifungal action of essential oils and phytochemical extracts against *Colletotrichum gloeosporioides* in mangoes: ultrastructural and GC-MS evidence

**DOI:** 10.3389/fmicb.2026.1853202

**Published:** 2026-06-25

**Authors:** Hardev Choudhary, Sunil Pareek, Varsha Dhar, Satya Singh, Vipin Kumar, Yasmeen Siddiqui, Asgar Ali

**Affiliations:** 1 Department of VARD & IPM-Agriculture, National Innovation Foundation – India, Gandhinagar, India; 2 Department of Agriculture and Environmental Sciences, National Institute of Food Technology, Entrepreneurship and Management, Sonepat, India; 3 Department of Business Development, National Innovation Foundation – India, Gandhinagar, India; 4 Department of Biological Sciences, College of Science, King Faisal University, Al Ahsa, Saudi Arabia; 5 Department of Integrative Agriculture, College of Agriculture and Veterinary Medicine, United Arab Emirates University, Al-Ain, United Arab Emirates

**Keywords:** anthracnose, antifungal, botanicals, *Mangifera indica*, MIC, phytopathogenic, postharvest management, secondary metabolites

## Abstract

**Introduction:**

Mango anthracnose, caused by *Colletotrichum gloeosporioides*, is a major pre- and post-harvest disease that reduces fruit quality, shelf life, and market value. Reliance on synthetic chemicals for postharvest disease management necessitates safer and sustainable alternatives due to health and environmental challenges.

**Methods:**

This study assessed the *in vitro* antifungal efficacy of six medicinal *plants-Anethum graveolens* (AG), *Ferula assa-foetida* (FA), *Justicia adhatoda* (JA), *Macrotyloma uniflorum* (MU), *Nyctanthes arbor-tristis* (NA), and *Vitex negundo* (VN)-using five solvent extracts and essential oils (EOs) against *C. gloeosporioides*. Gas Chromatography-Mass Spectrometry (GC-MS) identified bioactive compounds in the most effective phytoextracts, whereas Scanning Electron Microscopy (SEM) examined ultrastructural aberrations in fungal mycelia post-treatment. Statistical analyses (ANOVA and Bonferroni's post hoc test) assessed the effects of plant species, solvent polarity, and exposure duration on antifungal performance.

**Results:**

The results showed a clear dose-dependent inhibition of mycelial growth and spore production across treatments. EOs demonstrated the highest antifungal activity, achieving up to 100% inhibition at 40–80 μL mL^−1^, followed by petroleum ether (PE) extracts, whereas aqueous (AQ) and methanolic (MeOH) extracts were less effective. Among plants, AG showed the most consistent antifungal effect, followed by FA and JA. GC-MS profiling detected 137 compounds (84.6%−97.65% composition), including fatty acids (23.88%), terpenoids, sesquiterpene alcohols (11.48%), epoxides (8.58%), sulfides (7.11%), and terpenes (5.79%) known to disrupt fungal membranes and inhibit mycelial and spore growth.

**Discussion:**

The findings indicate that plant species are the key determinant of antifungal efficacy, whereas solvent type and exposure duration act as secondary modulators. EOs, particularly from AG and FA, show promise as eco-friendly alternatives to synthetic fungicides and could be integrated into sustainable strategies for managing mango anthracnose.

## 1 Introduction

Global food security is severely challenged by postharvest losses, which are estimated to claim up to 35% of fruits and vegetables (Tiwari et al., 2021). In mango (*Mangifera indica* L.), a climacteric fruit of major economic and nutritional importance, these losses are primarily caused by fungal pathogens during storage and transportation, posing a significant threat to production and trade (Le et al., 2022). Among plant pathogenic fungi, *Colletotrichum gloeosporioides* (Penz.) Penz. & Sacc. is the most destructive causal agent of anthracnose disease, responsible for up to half of all postharvest losses in tropical regions (Arauz, 2000; Alvindia and Acda, 2015; Ciofini et al., 2022). This hemibiotrophic fungus infects leaves, twigs, inflorescences, and especially fruit postharvest. After adhering to the fruit surface, spores germinate and form appressoria for penetration, often remaining quiescent until ripening (Choudhary et al., 2025). Upon ripening, rapid proliferation causes sunken, black necrotic lesions that drastically reduce quality, shelf life, and marketability (Dofuor et al., 2023).

For decades, anthracnose has been managed mainly with synthetic fungicides such as thiabendazole, benomyl, imazalil, prochloraz, and others (Arauz, 2000; Sundravadana et al., 2007; Alvindia and Mangoba, 2020). While effective, their overuse has led to resistance, food safety concerns, and regulatory restrictions. Some compounds, such as prochloraz and its metabolite 2,4,6-trichlorophenol (2,4,6-TCP), are carcinogenic and classified as priority pollutants by the US Environmental Protection Agency (Zheng et al., 2015; Fang et al., 2017). Moreover, sub-lethal fungicide doses can paradoxically enhance sporulation in *Colletotrichum* (Han et al., 2022). This reliance on chemicals underscores the need for safer, eco-friendly, and sustainable alternatives. Botanicals, particularly plant extracts and essential oils (EOs), are rich in bioactive secondary metabolites with broad-spectrum antifungal activity, biodegradability, and generally recognized as safe (GRAS) (Muniyappan et al., 2023; Leesutthiphonchai et al., 2024; Peralta-Ruiz et al., 2024) without harming beneficial organisms (Ramadwa et al., 2024) and preserving fruit quality (Alvindia and Mangoba, 2020). EOs, the secondary metabolites from aromatic plants, are complex mixtures of volatile, nonpolar, low molecular weight monoterpenes, sesquiterpenes, terpenoids, aromatic aldehydes, ketones, and alcohols, along with fatty acids and their esters (Shafi and Zahoor, 2020; Wu et al., 2023). Plant derivatives are an excellent source for developing antifungal agents, and studying their metabolic interactions can reveal microbial detoxification mechanisms and specific cellular targets, ensuring their effective and safe application (Chen et al., 2023).

Based on these considerations, six medicinal plants with documented antimicrobial potential were selected for this study: *Anethum graveolens* Roxb. (Apiaceae) (AG), *Ferula assa-foetida* L. (Ferulaceae) (FA), *Justicia adhatoda* L. (Acanthaceae) (JA), *Macrotyloma uniflorum* (Lam.) Verdc. (Fabaceae) (MU), *Nyctanthes arbor-tristis* Linn. (Oleaceae) (NA), and *Vitex negundo* L. (Lamiaceae) (VN). These species were chosen for their documented history of antimicrobial activity and abundance of bioactive constituents (Mohan et al., 2018; Ashokkumar et al., 2024; Islam et al., 2024). AG considered to have originated in Southwest Asia or Southeast Europe; is indigenous to the Mediterranean region, southern USSR, and Central Asia, and occurs in India, where it is widely cultivated for its foliage as a cool-season crop across the Indian subcontinent, the Malaysian archipelago, and Japan (Jana and Shekhawat, 2010; Ramadan et al., 2013; Al-Ibrahemi et al., 2022). It is renowned for its antioxidants and antimicrobial activities, primarily attributed to volatile compounds like carvone, limonene, α-phellandrene, and apiol, which also confer its distinctive aroma (Saleh-E-In and Choi, 2021; Wasim Akram et al., 2025). FA, a perennial herb, is predominantly distributed across the Mediterranean region and Central Asia, extending to North India and the Far East, and has been extensively used in traditional food systems and ethnomedicine, particularly in India, Afghanistan, Iran, and Mediterranean countries, where they are widely consumed (Bahetjan et al., 2023). Historically, FA has been recognized for its therapeutic applications in gastrointestinal disorders, infectious diseases, respiratory ailments, and rheumatoid arthritis (El Bitar et al., 2025). It produces a gum-resin rich in ferulic acid, sesquiterpene, coumarins, and sulfurous compounds, contributing to its broad pharmacological uses, including antimicrobial, antitumor, and neuroprotective effects (Amalraj and Gopi, 2017; Bahetjan et al., 2023; El Bitar et al., 2025). JA is a perennial shrub, traditionally used in Ayurvedic and Unani medicines and is widely distributed in the tropical regions of South and Southeast Asia, including the Indian subcontinent (Jayaweera et al., 2024; Mahajan et al., 2025). Its leaves are rich in phenols, alkaloids, tannins, flavonoids, quinazoline, and vasicinone, underpinning its use in treating respiratory ailments and its significant antimicrobial and hepatoprotective properties (Musa et al., 2022). MU, commonly called “horsegram,” is native to the Indian subcontinent and Africa (Fuller and Murphy, 2018; Oli et al., 2024). It is a highly nutritious legume containing several bioactive components such as phenolic acids (ferulic, vanillic acid), flavanols (kaempferol, quercetin), protease inhibitors, and phytic acid, which contribute to its documented therapeutic and antibacterial effects (Bhartiya et al., 2015; Prasad and Singh, 2015; Mohan et al., 2018; Padha et al., 2025). NA (Night jasmine or *Harsinghar*) is habituated in the subtropical Himalayas, Nepal, warm temperate regions of Europe, and Africa. Commonly found in tropical Asia, including India, Malaysia, and Indonesia, it is commonly cultivated in gardens (Ashokkumar et al., 2024). NA contains a diverse array of medicinal compounds across its tissues, including iridoid glycosides (arbortristosides A, B, C), nyctanthic acid, nyctanthoside-A, oleanolic acid, and various fatty acids, all of which are noted for their medicinal and antimicrobial potential (Venkataraman et al., 2019; Rani et al., 2023). It is a well-recognized, highly efficacious plant in Ayurveda, Homeopathy, Unani, and Siddha with stomachic, carminative, colon astringent, expectorant, anti-bilious, hair tonic, and therapeutic properties (Sharma et al., 2023; Ashokkumar et al., 2024). Finally, VN (chaste tree) is widely distributed across India, Sri Lanka, Madagascar, Malaysia, China, the Philippines, Vietnam, and East Africa (Van Vo et al., 2022; Venkatesan et al., 2024). It is a traditionally used medicinal plant whose pharmacological properties, including anti-inflammatory, antimicrobial, and hormonal regulatory effects, stem from a complex profile of flavonoids, iridoids, terpenes, steroids, and phenolic compounds such as caryophyllene epoxide, (E)-nerolidol, α-selinene, caffeic acid, and isochlorogenic acid (Huang et al., 2015; Nyamweya et al., 2023; Kar et al., 2024).

Although several plant species have previously been reported to possess antimicrobial properties, systematic comparative studies evaluating their bioefficacy against Anthracnose caused by *C. gloeosporioides* remain limited. In particular, there is insufficient understanding of how extraction solvents of different polarities influence the antifungal potential of plant-derived extracts in comparison with their essential oils (EOs). Since solvent polarity significantly affects phytochemical extraction, a comparative assessment of polar, semipolar, and nonpolar extracts is necessary to identify the most effective extraction systems and bioactive fractions against the pathogen. Furthermore, information on the phytochemical composition of potent extracts and EOs, their potential mechanisms of antifungal action against fungal mycelia, and their implications for the development of botanical fungicides remains fragmented. Such gaps restrict the scientific advancement and standardization of plant-based alternatives for sustainable postharvest disease management.

Therefore, the present study was undertaken to comparatively investigate the antifungal bioefficacy of extracts prepared using solvents of varying polarities, along with essential oils obtained from six selected plant species, against *C. gloeosporioides*. Specifically, the study aimed to:

1. Evaluate and compare the antifungal activity of polar, semi-polar, and nonpolar solvent extracts, and essential oils from the selected plants against *C. gloeosporioides*.
2. Examine ultrastructural alterations in fungal mycelia following treatment using Scanning Electron Microscopy (SEM).
3. Identify major bioactive constituents in the most effective extracts and EOs through Gas Chromatography–Mass Spectrometry (GC–MS) analysis to support future formulation-oriented research.

The broader objective of the study was to generate scientific evidence to support the development of plant-based, environmentally compatible antifungal agents as potential alternatives to synthetic fungicides for managing mango anthracnose.

## 2 Materials and methods

### 2.1 Culture collection and identification

The pure fungal strain (accession no. 8524) of *C. gloeosporioides*, isolated from mango, was procured from the Indian Type Culture Collection (ITCC) laboratory of the Indian Agricultural Research Institute, New Delhi, India. The strain was maintained on Potato Dextrose Agar (PDA) at 4 °C. To confirm the pathogenicity, healthy “Kesar” mango fruits were surface-sterilized using 1% sodium hypochlorite solution followed by 70% ethanol for 2–3 min. A 5-mm wound was created on each fruit using a cork borer, and inoculated with 100 μL of a spore suspension (1 x 10^5^ spores mL^−1^), and incubated. The inoculated fruits were stored in a humidified plastic container with sterile-distilled water-soaked cotton at 25 °C. The fruits were examined daily for the development of anthracnose. The pathogen was then re-isolated from the infected fruit, and its morphology and culture characteristics were confirmed to be the same as the original cultures. The culture was further identified at the Gujarat Biotechnology Research Center, Gandhinagar, Gujarat, India, using the Sanger sequencer-ABI 3500XL Genetic Analyzer (24 capillaries). The pure fungal culture was maintained by sub-culturing and stored at 4 °C for further use.

### 2.2 Plant material collection

Leaves of VN, JA, and NA were collected from 7-year-old plants cultivated in the herbal garden of the National Innovation Foundation–India (NIF), Gandhinagar, Gujarat (23°4′15.210′‘ N and 72°8′52.016^′′^ E), India. The seeds of MU and AG, and the gum resin of FA were procured from LVG Herbal Drugs Store, Ahmedabad, Gujarat, India. The samples of VN, JA, NA, AG, and MU were authenticated by matching the Virtual Herbarium records of the Botanical Survey of India with Barcodes BSID0016709, CAL0000025754, BSID0012365, BSI0000007313, and BSID0012796, respectively. These samples, including gum resin of FA, were also confirmed from the herbarium and crude drugs repository of NIF-India. Plant materials were washed under running water to remove dirt, insects, and debris, immediately shade-dried for 10 days, and further oven-dried at 40 ± 2 °C. The dried leaves were ground to a fine powder by using a laboratory pulverizer with a 2 mm sieve screen. The seeds of MU and AG were also dried at 40 ± 2 °C for 10 days and ground with a laboratory electric mixer to obtain fine powder using a 2 mm sieve screen. The gum resin of FA was ground by mortar and pestle into a coarse powder. The powder samples were packed in glass bottles with proper labels and stored in a modular cold room (RINAC-BEW & KC-407 CR/F) at 4 °C until used. EOs of the six species were sourced from Elite Biotech, Noida, Uttar Pradesh, India, and Globatic Herbs, Delhi, India.

### 2.3 Extraction of phytoextracts

Phytoextracts were prepared following the method described in the report (Azwanida, 2015) with slight modifications. Briefly, the coarsely powdered materials of *Ferula* (gum resin), fine powder of *Anethum* (seeds), *Macrotyloma* (seeds), *Justicia* (leaves), *Nyctanthes* (leaves), and *Vitex* (leaves) were macerated in five different analytical grade solvents purchased from Merck based on increasing polarity such as petroleum ether (>90%) (PE), chloroform (>99.5%) (TCM), ethyl acetate (>99.5%) (EA), methanol (>99.8%) (MeOH), and aqueous (double-distilled water) (AQ) with the ratio of 1:4 (w/v). The powder of each of the aforementioned plants (100 g) was dissolved in 400 mL of solvent at a ratio of 1:4 (w/v), i.e., 0.25 g mL^−1^. The maceration was carried out at ambient temperature (25 °C) for 72 h using a Thermo Scientific shaker (Model 481) at 150 × *g*. The extract was collected by filtering the crude mixture through a muslin cloth, followed by a Whatman filter paper (grade 1) using a vacuum filtration technique. The clear supernatant from all solvents was collected and dried to a concentrated extract at 40 °C on a magnetic stirrer with a hot plate in a fume hood. The aqueous extract was lyophilized to make a dried powder. The dried extract was weighed and stored at −4 °C until use. To prepare the desired 100 mg mL^−1^ (w/v) stock solutions, the dried extracts and EOs were diluted with 1% dimethyl sulfoxide (DMSO) on a weight-to-volume (w/v) basis and volume-to-volume (v/v) basis, respectively. Further, the obtained stocks were filtered through cellulose syringe filters (0.22 μm) and collected in amber glass vials and stored at −4 °C. The test concentrations were prepared from the stock solutions by further dilution with an appropriate volume of DMSO to achieve a final concentration of 20–80 μL mL^−1^.

### 2.4 *In vitro* antifungal bioassay

The antifungal bioefficacy of phytoextracts and EOs was tested against *C. gloeosporioides* using the poisoned food plate technique (Bhutia et al., 2016; Maqbool et al., 2010) with slight modification. *C. gloeosporioides* was cultured on PDA plates and incubated for 7 days at 25 °C to achieve optimal mycelial growth. A sterile 5-mm-diameter steel cork borer was used to punch mycelial disks from the actively growing fungal colonies, which served as the inoculum for the bioassay. Test concentrations of phytoextracts and Eos, ranging from 20–80 μL mL^−1^, were prepared. A 20 μL aliquot of each treatment concentration was pipetted onto the center of the surface of 60-mm diameter PDA petri plates. The plates were then allowed to stand for 5 min to ensure maximum diffusion of the extracts and EOs into the agar medium. A 5-mm diameter fungal disc was aseptically placed at the center of each poisoned PDA plate. The chemical fungicide azoxystrobin 23 SC (1 μL mL^−1^), neem oil (azadirachtin 0.15% w/w) at 5 μL mL^−1^ and 10 μL mL^−1^, and DMSO were used as positive and negative controls, respectively, to ensure the validity of results. The experiment was arranged in a Complete Randomized Design (CRD) with three replicates for each treatment and control. All plates were incubated at 25 °C for 5 days to ensure the fungal growth in the control plates reached the edges of the plate. The mycelial colony diameter was measured with a caliper on the 3rd and 5th days of incubation to assess the inhibitory effects of the treatments over time. Fungicidal activity was measured by determining the minimum inhibitory concentration (MIC_50_) required to inhibit 50% of mycelial growth. The percent inhibition of mycelial growth was determined by comparing the mycelial growth in DMSO-treated plates (negative control) using the following formula:


$$\begin{array}{l} PI=\frac{cd-td}{cd}\;\times 100 \end{array}$$

Where *PI* = mycelial growth inhibition; *cd* = mean diameter of mycelial growth of fungus of negative control plates; and *td* = mean diameter of the mycelial growth of the poisoned plates treated with different phytoextracts and EOs.

### 2.5 Spore count inhibition

The surfaces of *C. gloeosporioides* culture petri plates with different phytoextract treatments were flooded with 5 mL sterile distilled water. The surface was gently scraped using a sterile inoculating loop to remove the spores from the mycelium. Any fungal filaments were removed by filtering the spore suspension using a four-layered sterilized cheesecloth. The concentration of spores was adjusted to the desired level (1 x 10^5^ spores mL^−1^) using a hemocytometer before use. The spore count was performed under a light microscope by placing 10 μL spore suspension on each side of a hemocytometer (Chen and Dai, 2012). The following formula was used to calculate the inhibitory activity (IS) to spore formation.


$$\begin{array}{l} IS\;\left(\%\right)=\frac{dc-dt}{dc}\times 100 \end{array}$$

Where *IS* = inhibitory activity to the sporulation; *dc* = spore's density in negative control (DMSO); and *dt* = spore's density with treatments (phytoextracts and EOs).

### 2.6 Minimum inhibitory concentration bioassay

The MIC values were determined by the microbroth dilution method according to the guidelines of Clinical and Laboratory Standards Institute (Soria-Castro et al., 2019). The culture of *C. gloeosporioides* obtained from a 10-day-old plate was diluted to prepare a spore suspension. The colony surface was swabbed with sterile saline to harvest spores, which were allowed to settle for 10 to 15 min. The spore counts were standardized using a Neubauer chamber and adjusted to 1 × 10^5^ CFU mL^−1^ in RPMI medium. Based on the concentrations optimized for 50% radial growth inhibition using the poisoned food plate technique, phytoextracts ranging from 10–80 μL mL^−1^ (v/v) were also assessed to determine the MIC values against *C. gloeosporioides* in 96-well round-bottom microtiter plates. A 100 μL volume of antifungal solutions at different concentrations was added to each microplate well, followed by inoculation with 100 μL of a diluted *C. gloeosporioides*. The microplates were incubated at 25 °C without agitation for 24–48 h. The negative controls with fungus and medium were maintained accordingly. The positive control with antifungal suspension added to the medium, and the fungus was also included. The MIC values were determined at the lowest extract concentration at which fungal growth was absent. A 0.01% resazurin solution was applied to each well as a confirmation step for the color change. The MIC_50_ and MIC_90_ were determined by identifying the concentrations that caused exactly 50% and 90% inhibition of mycelial growth, respectively, on a PDA plate, based on the concentrations from each well that showed complete visual inhibition (Vargas Hernández et al., 2024).

### 2.7 Ultrastructural morphology study using scanning electron microscopy

The SEM testing was undertaken to find potential morphological abnormalities in *C. gloeosporioides* treated with effective phytoextracts and EOs adopted from Wu et al. (2023) with minor modifications. The samples of *C. gloeosporioides* were collected from the petri dishes with treatments and controls used in the antifungal study. The specimens of treated *C. gloeosporioides* were fixed in 4% glutaraldehyde for 3 h, followed by treatment with 0.1 M cacodylate buffer for 1 h. The specimens were washed with distilled water and dehydrated with ethanol (50%−100%) for 15 min each, followed by critical-point freeze-drying using a vacuum freeze-dyer. The specimens were mounted on an aluminum stub for gold coating. The specimen was observed under the SEM (FEI Nova Nano FEG-SEM 450) at an accelerating voltage of 20 kV.

### 2.8 Gas chromatography mass spectrometry analyses

The GC-MS analysis was conducted using a Clarus 680 GC and SQ8C MS system (PerkinElmer, Weltham, MA, USA), integrated with TurboMass software. The separation was carried out on an Elite 624 MS capillary column (30 m × 0.25 mm I.D. × 1.4 μm) from Perkin Elmer. The GC was set to operate at an oven temperature of 50 °C for 1 min, then heated from 50 °C to 250 °C at 10 °C min^−1^, finishing with a 10 min isothermal period. Helium served as the carrier gas. A 1 μL sample was injected at a split ratio of 1:50, and the injector temperature was maintained at 250 °C. The MS was used in electron-impact mode at 70 eV, scanning from 30 to 600 Da throughout 4 to 30 min. The data was collected and analyzed using TurboMass software. The phytoextracts and EO components were identified by their retention times and matched against the mass fragmentation patterns from the National Institute of Standards and Technology library. The chemical structures and IUPAC names were obtained from the PubChem database (https://pubchem.ncbi.nlm.nih.gov/).

### 2.9 Data analysis

All experiments were conducted in triplicate, and results were expressed as mean ± SD unless otherwise stated. The percent growth inhibition (GI) was also calculated. The data were subjected to suitable arcsine and square-root transformations. Three-way analysis of variance (ANOVA) was conducted to evaluate the effects of plant extracts × solvents × time on the mycelial growth. A 2-way ANOVA was also conducted to study the effects of EOs and time duration (3 days after treatment, DAT, and 5 DAT) on the mycelial growth and spore count. Pairwise comparisons of means were performed using Bonferroni's multiple comparisons test at *p* < 0.05. All the statistical analyses were performed using GraphPad Prism 10.5.0 (GraphPad Software, Boston, MA, USA).

## 3 Results

### 3.1 Extraction yields

All tested plant extracts and EOs inhibited the mycelial growth of *C. gloeosporioides* in a concentration-dependent manner. The net phytoextracts yield obtained from six medicinal plants ranged between 0.91% and 15.12% in five different solvents with increasing polarity (Table 1). JA leaves yielded the highest overall extraction percentages, particularly in AQ and MeOH with 15.12% and 13.67%, respectively. VA and NA showed their best yield with AQ (12.50% and 11.80%, respectively), followed by MeOH (10.25% and 10.06%, respectively). The AG-MeOH extract yielded the highest yield (4.70%), while AQ (2.9%) was the least effective. MU yielded slightly more in AQ (4.17%) than in MeOH (3.9%). Conversely, FA consistently produced the lowest yields across all solvents, with its maximum extraction occurring with AQ (2.56%) and MeOH (2.03%), highlighting its limited polar content. The nonpolar solvents consistently yielded lower yields for all plants, indicating a smaller quantity of lipophilic compounds or reduced extractability of these fractions.

**Table 1:** Total yield (%) obtained from various plants extracted in petroleum ether (PE), chloroform (TCM), ethyl acetate (EA), methanol (MeOH), and aqueous (AQ).

Plant	Yield (%)				
	PE	TCM	EA	MeOH	AQ
*Anethum graveolens* (seeds)	3.03	2.92	2.59	4.70	2.90
*Ferula assa-foetida* (gum resin)	0.91	1.11	1.17	2.03	2.56
*Justicia adhatoda* (leaves)	1.89	2.36	3.01	13.67	15.12
*Macrotyloma uniflorum* (seeds)	2.65	2.21	2.12	3.90	4.17
*Nyctanthes arbor-tristis* (leaves)	2.70	3.30	4.42	10.06	11.8
*Vitex negundo* (leaves)	3.20	3.50	3.91	10.25	12.50

### 3.2 *In vitro* antifungal bioassay

#### 3.2.1 Effects of different phytoextracts on the mycelial growth of *C. gloeosporioides*

**Figure 1:**
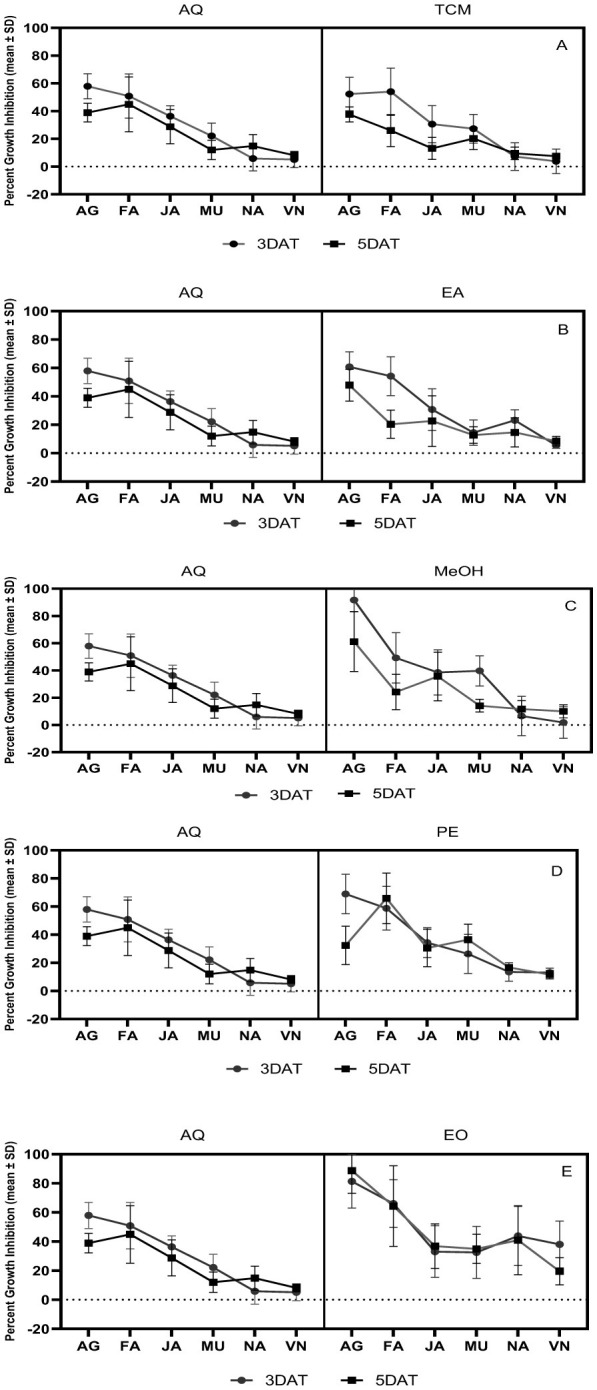
*In vitro* bioefficacy of different plant extracts in various solvents. AQ and TCM **(A)**, EA **(B)**, MeOH **(C)**, PE **(D)**, and EO **(E)** at different durations (3 DAT and 5 DAT) on the mycelial growth inhibition of *Colletorichum gloeosporioides*. AG, *A. graveolens;* FA, *F. assa-foetida;* JA, *J. adhatoda;* MU, *M. uniflorum;* NA, *N. arbor-tristis;* VN, *V. negundo;* AQ, aqueous extract; TCM, chloroform extract; EA, ethyl acetate extract; MeOH, methanol extract; PE, petroleum ether extract; EO, essential oil; DAT, days after treatment.

**Figure 2:**
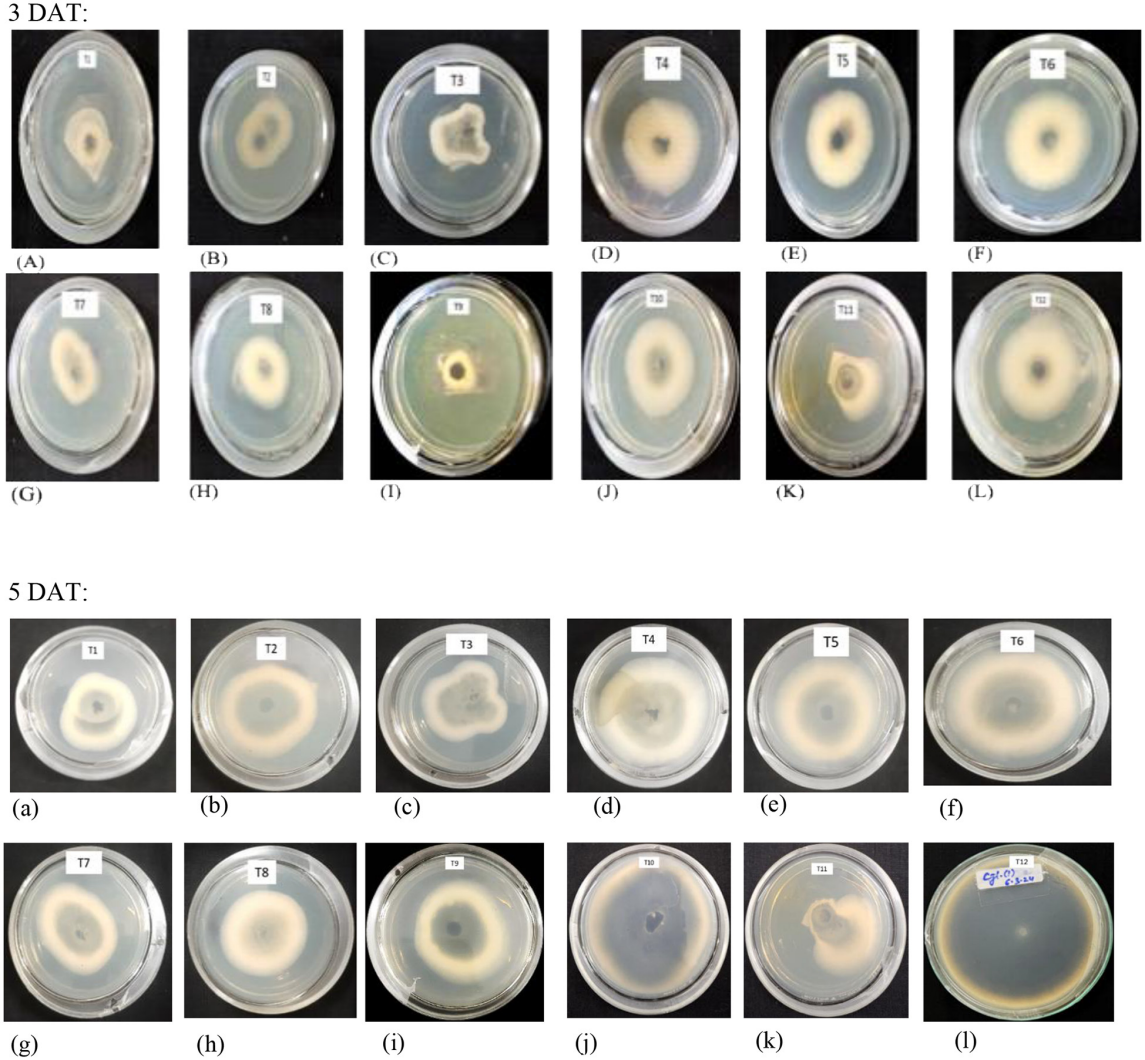
Images showing day-wise radial growth of *C. gloeosporioides* with different phytoextracts treatments on day 3 **(A–L)** and day 5 **(a–l)**. A, a: *Ferula* (PE); B, b: *Ferula* (AQ.); C, c: *Anethum* (MeOH.); D, d: *Justicia* (MeOH.); E, e: *Ferula* (EO); F, f: *Macrotyloma* (EO); G, g: *Anethum* (EA); H, h: *Anethum* (EO); I, i: *Nyctanthes* (EO); J, j: Neem; K, k: Azoxy; L, l: DMSO. AQ, aqueous extract; AZ, Azoxystrobin; TCM, chloroform extract; EA, ethyl acetate extract; MeOH, methanol extract; PE, petroleum ether extract; DAT, days after treatment; DMSO, dimethyl sulfoxide.

The results of the *in vitro* bioassay of phytoextracts show varying degrees of mycelial growth inhibition in *C. gloeosporioides* (Table 2). Among the various solvent extracts of AG, the best mycelial growth inhibition ranging between 78.54 ± 0.18% at 20 μL mL^−1^ to complete suppression (100.0 ± 0.00) at higher doses of 70 and 80 μL mL^−1^ was recorded in the methanol extract at 3 DAT followed by AG-PE extract (43.85% ± 0.18% to 83.47% ± 0.24%), AG-EA (47.43% ± 0.06% to 75.73% ± 0.18%), and AG-AQ extract (44.86% ± 0.12% to 70.14% ± 0.15%). The percent GI of AG-MeOH extract reduced at 5 DAT when compared with 3 DAT, ranging between 37.41% ± 0.13% at 20 μL mL^−1^ to 82.30% ± 0.13% and 93.26% ± 0.13% at 70 μL mL^−1^ and 80 μL mL^−1^, respectively (Table 2, Figures 1A–D; 2C, c). In case of FA, the PE extract provided the best inhibition of mycelial growth at 3 DAT (37.05% ± 0.12% to 81.25% ± 0.18%) followed by EA extract (35.73% ± 0.18% to 72.53% ± 0.24%), while at 5 DAT the percent GI was in the range of 48.96% ± 0.13% to 90.40% ± 0.04% in case of PE extract followed by 16.52% ± 0.13% to 71.19% ± 0.13% in AQ extract (Table 2, Figures 1A–D; 2A, a, B, b). For JA extracts, moderate efficacy in inhibiting mycelial growth was observed across all the solvents. The JA-MeOH extract, at 3 DAT, caused 21.15 ± 0.36% reduction in GI at 20 μL mL^−1^ which increased to 63.23% ± 0.18% at 80 μL mL^−1^, whereas in case of 5 DAT the efficacy ranged between 15.23% ± 0.09% to 60.40% ± 0.04% at 20–80 μL mL^−1^ doses (Table 2, Figures 1A–D; 2D, d). Methanol extract of MU at 3 DAT, with efficacy ranging between 22.99% ± 0.12% to 51.11% ± 0.12%, and PE extract at 5 DAT with 23.93% ± 0.06% to 48.96% ± 0.13% reduction in mycelial growth at 20–80 μL mL^−1^ (Table 2, Figures 1A–D; 2F) provided moderate efficacy against *C. gloeosporioides*. The extracts of NA and VN in all selected solvents were less effective, yielding low efficacy (Table 2, Figures 1A–D).

**Table 2:** *In vitro* bioefficacy of different plants extracted in polar and nonpolar solvents on the mycelial growth inhibition of *Colletotrichum gloeosporioides* at varying time durations.

Extracts	Percent mycelial growth inhibition (Mean ±SD)										
	Solvents	AQ		TCM		EA		MeOH		PE	
	Conc. (μLmL^−1^)	3 DAT	5 DAT	3 DAT	5 DAT	3 DAT	5 DAT	3 DAT	5 DAT	3 DAT	5 DAT
AG	20	44.86 ± 0.12aA	28.40 ± 0.43aA	33.85 ± 0.18aB	31.70 ± 0.06aA	47.43 ± 0.06aA	33.85 ± 0.13aA	78.54 ± 0.18aC	37.41 ± 0.13aB	43.85 ± 0.18aA	10.59 ± 0.06aC
	30	50.10 ± 0.18aA	30.86 ± 0.21aA	42.08 ± 1.44aA	32.30 ± 0.13aA	50.56 ± 0.06aA	37.85 ± 0.13aA	85.73 ± 0.18aB	40.07 ± 0.13bB	57.36 ± 0.12bA	16.74 ± 0.13bC
	40	55.80 ± 0.12bA	40.07 ± 0.13bA	46.18 ± 0.60bB	32.69 ± 0.17aA	54.79 ± 0.18aA	41.58 ± 0.17aA	87.29 ± 0.18bC	43.80 ± 0.17cB	66.74 ± 0.12cD	32.52 ± 0.13cC
	50	55.99 ± 0.00cA	41.73 ± 0.43cA	53.23 ± 0.18cA	38.25 ± 0.17aA	60.10 ± 0.18bA	46.94 ± 0.15bA	90.73 ± 0.18cB	60.79 ± 0.09dB	75.10 ± 0.18dC	37.46 ± 0.09dA
	60	62.05 ± 0.12dA	41.98 ± 0.21dA	61.04 ± 0.18dA	41.38 ± 0.15bA	65.10 ± 0.18cA	51.19 ± 0.13cB	99.17 ± 0.18dB	70.47 ± 0.17eC	76.11 ± 0.12eC	39.19 ± 0.13eA
	70	66.74 ± 0.12eA	43.70 ± 0.64eA	64.17 ± 0.18eA	43.09 ± 0.21cA	71.35 ± 0.18dA	58.96 ± 0.13dB	100.0 ± 0.00eB	82.30 ± 0.13fC	79.79 ± 0.18fC	43.41 ± 0.13fA
	80	70.14 ± 0.15fA	46.05 ± 0.53fA	65.73 ± 0.18fA	44.12 ± 0.00dA	75.73 ± 0.18eA	64.52 ± 0.13eB	100.0 ± 0.00fB	93.26 ± 0.13gC	83.47 ± 0.24gC	46.64 ± 0.02gA
FA	20	24.27 ± 0.09aA	16.52 ± 0.13aA	29.24 ± 0.12aA	10.79 ± 0.09aB	35.73 ± 0.18aB	8.25 ± 0.17aC	21.98 ± 0.18aC	7.85 ± 0.13aD	37.05 ± 0.12aD	40.96 ± 0.13aE
	30	38.70 ± 0.05bA	28.52 ± 0.13bA	38.61 ± 0.12bA	15.23 ± 0.09aB	41.09 ± 0.14aA	10.07 ± 0.13aC	31.69 ± 0.16aB	11.90 ± 0.09bD	44.31 ± 0.06aA	48.96 ± 0.13bE
	40	47.99 ± 0.12cA	33.26 ± 0.13cA	47.43 ± 0.06cA	19.68 ± 0.09bB	47.60 ± 0.18bA	14.84 ± 0.04bC	42.07 ± 0.10bB	18.25 ± 0.17cD	51.67 ± 0.18bA	57.46 ± 0.09cE
	50	53.70 ± 0.05dA	47.06 ± 0.04dA	53.78 ± 0.24dA	26.74 ± 0.13cB	53.68 ± 0.06cA	20.47 ± 0.17cC	51.11 ± 0.12cB	22.69 ± 0.17dD	60.21 ± 0.09cA	66.74 ± 0.13dE
	60	55.52 ± 0.09eA	54.51 ± 0.13eA	62.05 ± 0.12eA	30.79 ± 0.09dB	61.04 ± 0.18dA	24.52 ± 0.13dC	58.81 ± 0.22dB	29.36 ± 0.17eD	64.90 ± 0.09dC	74.12 ± 0.09eE
	70	63.33 ± 0.09fA	63.41 ± 0.13fA	69.86 ± 0.12fA	36.02 ± 0.17eB	67.74 ± 0.06eA	29.68 ± 0.09eC	65.73 ± 0.18eB	35.63 ± 0.13fD	71.88 ± 0.18eC	82.62 ± 0.04fE
	80	72.71 ± 0.09gA	71.19 ± 0.13gA	77.12 ± 0.06gA	43.01 ± 0.09fB	72.53 ± 0.24fA	34.91 ± 0.17fC	73.32 ± 0.10fB	43.80 ± 0.17gD	81.25 ± 0.18fC	90.40 ± 0.04gE
JA	20	30.24 ± 0.06aA	17.85 ± 0.13aA	12.60 ± 0.18aB	2.81 ± 0.06aB	8.92 ± 0.12aC	4.84 ± 0.04aC	21.15 ± 0.36aD	15.23 ± 0.09aA	22.08 ± 0.36aE	18.57 ± 0.09aA
	30	30.52 ± 0.09aA	18.57 ± 0.09aA	16.74 ± 0.12aB	6.35 ± 0.09bB	17.74 ± 0.06bC	7.06 ± 0.04aC	22.92 ± 0.18aD	18.57 ± 0.09aA	23.23 ± 0.18aE	20.07 ± 0.13aA
	40	31.35 ± 0.18aA	20.07 ± 0.13aA	26.11 ± 0.12bA	9.28 ± 0.04cB	26.67 ± 0.18cA	10.07 ± 0.13bC	27.29 ± 0.18aB	24.52 ± 0.13bD	28.23 ± 0.18aazA	21.19 ± 0.13aA
	50	33.92 ± 0.12aA	24.12 ± 0.09bA	29.24 ± 0.12cA	12.30 ± 0.13dB	29.79 ± 0.18dA	18.17 ± 0.04cC	33.54 ± 0.18bA	30.42 ± 0.02cD	35.49 ± 0.12bA	22.30 ± 0.13aA
	60	35.49 ± 0.12aA	30.79 ± 0.09cA	38.06 ± 0.06dA	15.23 ± 0.09eB	37.05 ± 0.12eA	25.23 ± 0.09dC	46.35 ± 0.18cA	46.74 ± 0.13dD	38.06 ± 0.06cA	35.23 ± 0.09bA
	70	42.29 ± 0.18bA	39.68 ± 0.09dA	42.74 ± 0.06eA	20.79 ± 0.09fB	43.85 ± 0.18fA	42.62 ± 0.04eA	55.42 ± 0.18dB	53.43 ± 0.11eC	42.29 ± 0.18dA	47.06 ± 0.04cD
	80	50.56 ± 0.06cA	50.40 ± 0.04eA	48.99 ± 0.06fA	25.23 ± 0.09gB	51.11 ± 0.12gA	50.40 ± 0.04fA	63.23 ± 0.18eA	60.40 ± 0.04fC	51.11 ± 0.12eA	49.68 ± 0.09dA
MU	20	11.04 ± 0.18aA	3.93 ± 0.06aA	18.02 ± 0.09aB	12.30 ± 0.13aB	2.40 ± 0.09aC	5.23 ± 0.09gA	22.99 ± 0.12aA	8.17 ± 0.04aC	10.21 ± 0.09aA	23.93 ± 0.06aD
	30	14.17 ± 0.18aA	5.23 ± 0.09aA	19.86 ± 0.12aA	13.73 ± 0.04aB	5.80 ± 0.12bB	8.17 ± 0.04aA	26.11 ± 0.12bA	9.48 ± 0.06aC	13.61 ± 0.12aA	24.84 ± 0.04aD
	40	15.73 ± 0.18aA	7.19 ± 0.13bA	21.98 ± 0.18bB	13.73 ± 0.04bB	10.49 ± 0.12cC	8.96 ± 0.13bA	39.17 ± 0.18cD	12.62 ± 0.04bC	15.73 ± 0.18aA	26.74 ± 0.13aD
	50	20.42 ± 0.18aA	12.52 ± 0.13cA	22.43 ± 0.06cA	18.17 ± 0.04cB	15.17 ± 0.12dA	12.30 ± 0.13cA	44.31 ± 0.06dC	14.12 ± 0.09cC	25.56 ± 0.06bA	38.96 ± 0.13bD
	60	26.11 ± 0.12bA	13.73 ± 0.04dA	25.56 ± 0.06dA	23.01 ± 0.09dB	16.74 ± 0.12eB	15.23 ± 0.09dA	46.98 ± 0.18eC	15.23 ± 0.09dB	33.37 ± 0.06cD	44.52 ± 0.13cC
	70	32.36 ± 0.12bA	19.68 ± 0.09eA	38.06 ± 0.06eA	27.46 ± 0.09eB	24.55 ± 0.12fB	18.17 ± 0.04eA	47.43 ± 0.06fC	18.17 ± 0.04eC	40.73 ± 0.18dD	47.06 ± 0.04dD
	80	34.93 ± 0.06dA	21.90 ± 0.09fA	45.42 ± 0.18fB	33.01 ± 0.09fB	26.11 ± 0.12gC	21.19 ± 0.13fA	51.11 ± 0.12gD	21.19 ± 0.13fC	45.42 ± 0.18eE	48.96 ± 0.13eD
NA	20	−6.15 ± 0.18aA	2.74 ± 0.13aA	−11.39 ± 0.12aB	3.73 ± 0.04aA	11.35 ± 0.18aB	2.74 ± 0.13aA	−12.40 ± 0.18aC	3.93 ± 0.06aB	3.68 ± 0.06aA	14.12 ± 0.09aC
	30	−0.90 ± 0.24bA	4.52 ± 0.13aA	2.12 ± 0.06bA	4.52 ± 0.13aA	16.98 ± 0.18aB	5.23 ± 0.09aA	−10.10 ± 0.09aC	4.84 ± 0.04aB	6.15 ± 0.18aD	14.52 ± 0.13aC
	40	2.67 ± 0.12aA	14.84 ± 0.04bA	5.24 ± 0.06cA	8.37 ± 0.06bB	20.87 ± 0.06bB	8.17 ± 0.04bC	3.23 ± 0.18bC	10.04 ± 0.06bD	13.06 ± 0.06bD	15.63 ± 0.13aA
	50	4.79 ± 0.18aA	16.74 ± 0.13cA	9.48 ± 0.18aB	10.07 ± 0.13cB	22.99 ± 0.12cC	13.80 ± 0.17cA	4.79 ± 0.18cD	11.90 ± 0.09cC	14.17 ± 0.18cE	17.06 ± 0.04aA
	60	5.80 ± 0.12aA	18.57 ± 0.09dA	10.49 ± 0.12aB	10.59 ± 0.06dB	27.60 ± 0.18dC	17.65 ± 0.11dA	16.35 ± 0.18dD	12.62 ± 0.04dC	17.29 ± 0.18dE	17.85 ± 0.13aA
	70	16.18 ± 0.06cA	23.01 ± 0.09eA	16.98 ± 0.18aA	13.41 ± 0.13eB	30.10 ± 0.18eB	23.73 ± 0.04eA	20.42 ± 0.18eC	19.68 ± 0.09eB	18.30 ± 0.12eA	18.57 ± 0.09aA
	80	18.85 ± 0.18dA	23.21 ± 0.11fA	17.74 ± 0.06dA	16.15 ± 0.06fB	31.98 ± 0.18fB	31.19 ± 0.13fA	23.99 ± 0.06fC	18.96 ± 0.13fC	21.98 ± 0.18fA	19.28 ± 0.04aA
VN	20	−2.33 ± 0.12aA	4.07 ± 0.13aA	−13.89 ± 1.20aB	4.84 ± 0.04aA	3.19 ± 0.13aA	4.36 ± 0.05aA	−16.63 ± 0.06aC	3.41 ± 0.13aB	9.48 ± 0.18aD	7.46 ± 0.09aC
	30	1.11 ± 0.12aA	4.84 ± 0.04aA	1.11 ± 0.12bA	5.23 ± 0.09aA	5.23 ± 0.09aB	4.27 ± 0.08bA	−9.27 ± 0.18bC	4.52 ± 0.13aB	10.21 ± 0.09aD	9.28 ± 0.04aC
	40	2.67 ± 0.12aA	8.17 ± 0.04bA	3.68 ± 0.06cA	6.74 ± 0.13aA	7.46 ± 0.09aA	4.17 ± 0.14cA	1.11 ± 0.12cB	8.57 ± 0.09bB	11.04 ± 0.18aC	9.68 ± 0.09aA
	50	3.68 ± 0.06aA	8.81 ± 0.13cA	4.24 ± 0.12dA	7.46 ± 0.09aA	9.68 ± 0.09aA	4.07 ± 0.18dA	1.67 ± 0.18dB	10.79 ± 0.09cB	12.60 ± 0.18aC	11.90 ± 0.09bA
	60	5.24 ± 0.06aA	9.68 ± 0.09dA	9.48 ± 0.18aA	8.57 ± 0.09bA	10.79 ± 0.09aA	4.02 ± 0.12eA	8.37 ± 0.06eB	12.30 ± 0.13dB	14.17 ± 0.18aC	13.41 ± 0.13cA
	70	12.05 ± 0.12bA	10.40 ± 0.04eA	11.04 ± 0.18aA	9.68 ± 0.09cA	11.19 ± 0.13aB	4.0 ± 0.15fB	11.49 ± 0.06fC	14.84 ± 0.04eB	14.90 ± 0.09aA	14.12 ± 0.09dA
	80	13.61 ± 0.12cA	10.91 ± 0.02fA	11.49 ± 0.18aA	10.07 ± 0.13dA	11.90 ± 0.09bA	3.97 ± 0.26gB	16.18 ± 0.06gB	15.63 ± 0.13fA	18.85 ± 0.18bA	15.63 ± 0.13eA
NO	5	20.52 ± 0.18cA	5.63 ± 0.13aA	20.52 ± 0.18aA	5.63 ± 0.13aA	20.52 ± 0.18cA	5.63 ± 0.13hA	20.52 ± 0.18hA	5.63 ± 0.13gA	20.52 ± 0.18cE	5.63 ± 0.13fA
AZ	1	76.67 ± 0.36dA	75.63 ± 0.13gA	76.67 ± 0.36eA	75.63 ± 0.13eA	76.67 ± 0.36dA	75.63 ± 0.13iA	76.67 ± 0.36iA	75.63 ± 0.13hA	76.67 ± 0.36dA	75.63 ± 0.13gA
DMSO	1%	0.00aA	0.00aA	0.00aA	0.00aA	0.00aA	0.00aA	0.00aA	0.00aA	0.00aA	0.00aA

Values are mean ± SD; n = 3. AG, *A. graveolens*; FA, *F. assa-foetida*; JA, *J. adhatoda*; MU, *M. uniflorum*; NA, *N. arbor-tristis*; VN, *V. negundo*; AQ, aqueous extract; AZ, Azoxystrobin; TCM, chloroform extract; EA, ethyl acetate extract; MeOH, methanol extract; PE, petroleum ether extract; DAT, days after treatment; DMSO, dimethyl sulfoxide; SD, standard deviation; NO, neem oil. Uppercase letters within a row indicate significant differences among solvents at the same dose at different time durations (3 DAT & 5 DAT), whereas lowercase letters within a column indicate significant differences among doses within the same solvent according to Bonferroni's multiple comparison test (α = 0.05).

Three-way ANOVA was performed to assess the effects of plant, solvent type, and time on the percentage growth inhibition of *C. gloeosporioides* mycelia with solvent as the matching factor. The analysis revealed that all main effects and their interactions were statistically significant (*p* < 0.05), indicating that plant, solvent type, and time post-treatment all significantly influenced antifungal activity, both independently and interactively (Table 3).

**Table 3:** Summary of comparison of the effects of different plants extracted in various solvents at various time durations (3 DAT vs. 5 DAT) on the mycelial growth inhibition of *Colletotrichum gloeosporioides*, tested by using three-way ANOVA followed by Bonferroni's multiple comparisons.

Solvents	AQ					TCM					EA					ME					PE				
Source of interaction	SS	DF	MS	F	*p-value*	SS	DF	MS	F	*p-value*	SS	DF	MS	F	*p-value*	SS	DF	MS	F	*p-value*	SS	DF	MS	F	*p-value*
A	72,453	8	9057	154.8	< 0.0001	85,596	8	10700	182.9	< 0.0001	74,084	8	9,260	142	< 0.0001	88,424	8	11,053	93.3	< 0.0001	77,085	8	9636	146	< 0.0001
B	62.72	1	62.72	18.65	< 0.0001	62.72	1	62.72	18.65	< 0.0001	10.6	1	10.6	2.19	0.1421	7,146	1	7,146	455	< 0.0001	702	1	702	162	< 0.0001
C	1,543	1	1543	26.38	< 0.0001	2612	1	2612	44.65	< 0.0001	1,411	1	1,411	21.7	< 0.0001	560	1	560	4.73	0.0319	630	1	630	9.55	0.0025
A × B	757.2	8	94.64	28.14	< 0.0001	757.2	8	94.64	28.14	< 0.0001	894	8	112	23.1	< 0.0001	7,497	8	937	59.7	< 0.0001	929	8	116	26.8	< 0.0001
A × C	2,033	8	254.1	4.34	0.0001	2,963	8	370.3	6.33	< 0.0001	1,926	8	241	3.7	< 0.0001	1,122	8	140	1.18	0.3159	2,822	8	353	5.35	< 0.0001
B × C	129.8	1	129.8	38.58	< 0.0001	129.8	1	129.8	38.58	< 0.0001	95.4	1	95.4	19.7	< 0.0001	17.1	1	17.1	1.09	0.2989	7.34	1	7.34	1.69	0.1961
A × B × C	438.4	8	54.8	16.29	< 0.0001	438.4	8	54.8	16.29	< 0.0001	894	8	112	23.1	< 0.0001	1,052	8	132	8.38	< 0.0001	792	8	99	22.8	< 0.0001

A = Plant (*A. graveolens, F. assa-foetida, J. adhatoda, M. uniflorum, N. arbor-tristis, and V. negundo*); B = solvents (Aqueous, Chloroform, Ethyl acetate, Methanol and Petroleum ether); C = Time (3 DAT & 5 DAT); AQ, aqueous extract, TCM, chloroform extract, EA, ethyl acetate extract, ME, methanol extract, PE, petroleum ether extract, DAT, days after treatment, SS, sum of squares; Df, degree of freedom; MS, mean square; F, fisher value; P, probability.

The plant species was the most influential factor, accounting for 86.25% of the total variation [*F*_(8, 108)_ = 182.9, *p* < 0.0001] in TCM extract and AQ extract [86.20% variation, *F*_(8, 108)_ = 155, *p* < 0.0001]. Solvent in both AQ [0.074%, *F*_(1, 108)_ = 18.9, *p* < 0.0001] and TCM [0.063%; *F*_(1, 108)_ = 18.65, *p* < 0.0001] were significant, although their contributions to total variation were comparatively small. Similarly, significant yet low total variation effects were also reported for the time factor for TCM [2.63%, *F*_(1, 108)_ = 44.65, *p* < 0.0001] and AQ [1.83%, *F*_(1, 108)_ = 26.4, *p* < 0.0001], respectively. Significant interactions were observed for plant × solvent [*F*_(8, 108)_ = 28.14(AQ) and *F*_(8, 108)_ = 28.14 (TCM), *p* < 0.0001], plant × time [*F*_(8, 108)_ = 4.36 (AQ), *p* = 0.0001; *F*_(8, 108)_ = 6.33 (TCM), *p* < 0.0001], solvent × time [*F*_(1, 108)_ = 39.8 (AQ), *F*_(1, 108)_ = 38.58 (TCM), *p* < 0.0001], and the three-way interaction plant × solvent × time [*F*_(8, 108)_ = 16.6 (AQ) and *F*_(8, 108)_ = 16.29, (TCM), *p* < 0.0001; Table 3]. The *post hoc* Bonferroni's multiple comparisons test showed that AG exhibited significantly greater inhibition compared with JA, MU, NA, VN, and neem (*p* < 0.0001). AG also differed markedly from the negative control DMSO (*p* < 0.0001), confirming its superior antifungal potency. No significant difference was observed between AG and FA (*p* > 0.9999). Conversely, azoxystrobin, the chemical control, was significantly more effective than AG (*t* = 8.66, *p* < 0.0001).

In the case of EA, the analysis revealed that the plant factor accounted for the largest proportion of variation [85.30%, *F*_(8, 108)_ = 142.0, *p* < 0.0001], indicating highly significant differences among the tested plant extracts. Conversely, the solvent effect was not significant [*F*_(1, 108)_ = 2.19, *p* = 0.1421], suggesting that the choice of solvent did not influence the activity independently. The factor of time also had a significant effect [1.62%, *F*_(1, 108)_ = 21.70, *p* < 0.0001; Table 3], though its contribution to total variance was relatively small. Significant two-way interactions were observed between plant × solvent (*p* < 0.0001), plant × time (*p* = 0.0007), and solvent × time (*p* < 0.0001; Table 3), highlighting that the inhibitory effect of extracts varied depending on both solvent type and duration of exposure. Importantly, a three-way interaction (plant × solvent × time) was also significant [*F*_(8, 108)_ = 23.1, *p* < 0.0001], indicating that the growth inhibitory effect was jointly influenced by all three factors (Table 3). *Post hoc* Bonferroni comparisons demonstrated that AG differed significantly from most other plant extracts. While differences between AG and FA were not significant (*p* = 0.0898), AG showed significantly higher inhibitory activity compared to JA, MU, NA, VN, and neem (*p* < 0.0001).

In the case of MeOH, plant extracts were the dominant source of variation, accounting for 73.5% of the total variance [*F*_(8, 108)_ = 93.3, *p* < 0.0001]. This indicates substantial inherent differences among plant extracts in their bioefficacy. Solvent type also exerted a significant effect, accounting for 5.94% of the total variance [*F*_(1, 108)_ = 455, *p* < 0.0001], demonstrating that the effect of extraction medium-MeOH significantly influenced the growth inhibition. Conversely, time exerted a low significant effect on percent growth inhibition [*F*_(1, 108)_ = 4.73, *p* = 0.0319], accounting for only 0.46% of the total variation, suggesting that overall inhibition levels were comparable between the two observation periods (Table 3). The binary interactions between plant × solvent were highly significant [*F*_(8, 108)_ = 59.7, *p* < 0.0001], accounting for 6.23% of the variance, indicating that the effect of solvent differed markedly among plants. The three-way interaction (Plant × Solvent × Time) was also significant [*F*_(8, 108)_ = 8.38, *p* < 0.0001]. However, it explained only a small proportion of variance (0.875%), suggesting that the changes in percent growth inhibition depended on specific plant–solvent combinations. Conversely, Plant × Time and Solvent × Time interactions were not significant (*p* > 0.05), indicating that temporal effects were generally consistent across plants and solvents when considered independently (Table 3). *Post hoc* comparisons with Bonferroni's multiple comparisons test showed that AG exhibited significantly higher growth inhibition compared with FA (*p* = 0.0031), JA (*p* < 0.0001), MU (*p* < 0.0001), NA (*p* < 0.0001), VN (*p* < 0.0001), and neem (*p* < 0.0001). AG also differed significantly from DMSO (*p* < 0.0001). Conversely, no significant difference was found between AG and azoxystrobin (*p* > 0.9999), suggesting comparable efficacy (Table 3).

Similar results were observed in the PE extract, where the plant was the dominant factor, explaining 85.1% of the total variation [*F*_(8, 108)_ = 146.0, *p* < 0.0001], indicating big differences among species. Solvent also had a significant effect [0.775% of variation, *F*_(1, 108)_ = 162, *p* < 0.0001; Table 3]. Time accounted for only 0.695% of variation and was also significant [*F*_(1, 108)_ = 9.55, *p* = 0.0025]. Significant interactions were observed for plant × solvent [*F*_(8, 108)_ = 26.8, *p* < 0.0001], plant × time [*F*_(8, 108)_ = 5.35, *p* < 0.0001], solvent × time [*F*_(1, 108)_ = 1.69, *p* = 0.1961], and the three-way interaction plant × solvent × time [*F*_(8, 108)_ = 22.8, *p* < 0.0001; Table 3], suggesting that the effect of plant extracts depended on both the solvent used and exposure duration. *Post hoc* comparisons showed no significant difference between AG and FA (*p* = 0.9207). AG showed significantly higher percent GI compared with JA (*t* = 4.71, *p* < 0.0001), MU (*t* = 7.43, *p* < 0.0001), NA (*t* = 11.2, *p* < 0.0001), VN (*t* = 12.5, *p* < 0.0001), and neem (*t* = 11.2, *p* < 0.0001). Compared with azoxystrobin, AG exhibited lower inhibition (*t* = 7.4, *p* < 0.0001), indicating that azoxystrobin was more effective.

**Figure 3:**
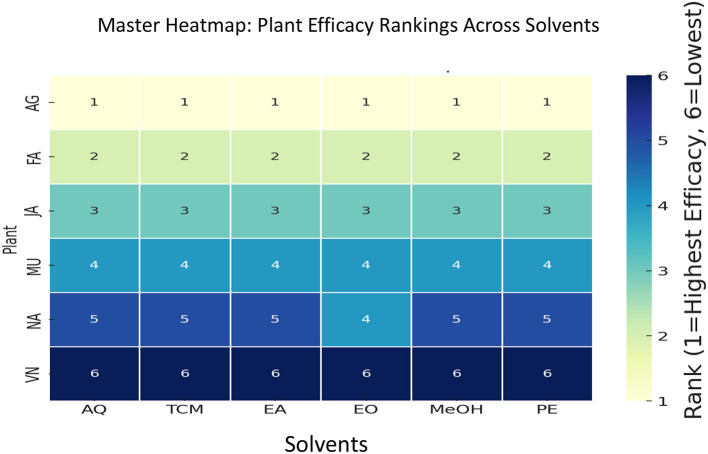
Comparative antifungal efficacy ranking of six medicinal plant extracts across six solvent systems (AQ, TCM, EA, EO, MeOH, PE). Ranking values (1–6) represent relative antifungal performance within each solvent, where 1 indicates the highest inhibition and 6 the lowest. *Anethum graveolens* (AG) consistently ranked highest, followed by *Ferula assa-foetida* (FA) and *Justicia adhatoda* (JA), whereas *Macrotyloma uniflorum* (MU), *Nyctanthes arbor-tristis* (NA), and *Vitex negundo* (VN) showed comparatively lower antifungal activity. AQ, aqueous extract; TCM, chloroform extract; EA, ethyl acetate extract; EO, essential oil; MeOH, methanol extract; PE, petroleum ether extract.

Across all plant and solvent combinations, AG consistently achieved the highest rank, confirming its robust and reproducible antifungal efficacy. FA followed closely in second place, while JA occupied a stable mid-tier (third rank) across treatments. Conversely, MU, NA, and VN clustered in the lower efficacy tier, with *Vitex* consistently occupying the last position. Occasional solvent-specific variations were noted, such as NA aligning with *Macrotyloma* under certain extracts (e.g., aqueous vs. EO), but these shifts did not alter overall ranking patterns. Statistical comparisons further reinforced these trends: AG and FA displayed highly significant superiority (*p* < 0.0001) across all solvents, JA showed moderate significance (*p* < 0.002) over some lower-ranked species, while differences among MU, NA, and VN were often not significant, consistent with their grouping within the lowest efficacy tier (Figure 3).

#### 3.2.2 Effects of EOs on mycelial growth of *C. gloeosporioides*

EOs from different plants inhibited the radial growth of *C. gloeosporioides* mycelia to varying degrees (< 50% = low, 50%−75% = moderate, and >75% = high). The EOs of AG and FA reported strong suppression in mycelial growth from 30 μL mL^−1^ to 80 μL mL^−1^ doses, with complete suppression at ≥60 μL mL^−1^ maintained by AG (Tables 4, 5, Figures 1E, 2H, h, E, e). However, the phytoextracts of JA-MeOH (Figures 2D, d), MU-EO (Figures 2F, f), NA-EO (Figures 2I, i), AG-EA (Figures 2G, g), NO (Figures 2J, j), and VN-EO (Table 4) retained low-to-moderate dose-dependent fungistatic activity at both 3 and 5 DAT, suggesting prolonged bioefficacy. Chemical control AZ was superior to phytoextracts in inhibiting the mycelial growth (Figures 2K, k). From 3 DAT to 5 DAT, a time-dependent decline in efficacy across most EOs was observed (Table 4, Figure 1E), except AG EO, which performed consistently over time.

**Table 4:** *In vitro* bioefficacy of different plant essential oils on the mycelial growth inhibition of *Colletotrichum gloeosporioides* at varying time durations.

	Mycelial growth inhibition (Percent ±SD)											
Conc. (μLmL^−1^)	AG_EO		FA_EO		JA_EO		MU_EO		NA_EO		VN_EO	
	3 DAT	5 DAT	3 DAT	5 DAT	3 DAT	5 DAT	3 DAT	5 DAT	3 DAT	5 DAT	3 DAT	5 DAT
20	53.96 ± 0.09aA	61.63 ± 0.13aA	43.30 ± 0.12aB	26.02 ± 0.17aB	13.68 ± 0.06aC	17.49 ± 0.05aC	8.23 ± 0.18aD	23.73 ± 0.04aD	20.87 ± 0.06aE	14.84 ± 0.04aE	13.33 ± 0.09aF	8.96 ± 0.13aF
30	62.60 ± 0.18aA	73.41 ± 0.13bA	51.67 ± 0.18aB	36.15 ± 0.06bB	15.73 ± 0.18aC	20.65 ± 0.02aC	14.48 ± 0.18D	27.85 ± 0.13aD	23.99 ± 0.06bE	17.93 ± 0.26aE	23.99 ± 0.06aF	10.37 ± 0.00aF
40	74.10 ± 0.24bA	87.19 ± 0.13cA	58.37 ± 0.06bB	52.30 ± 0.13cB	20.42 ± 0.18bC	29.28 ± 0.04bC	23.54 ± 0.18cD	29.48 ± 0.06aD	30.24 ± 0.06cE	23.63 ± 0.13bE	31.81 ± 0.06aF	13.73 ± 0.04bF
50	84.48 ± 0.18cA	99.04 ± 0.13gA	65.17 ± 0.12cB	66.02 ± 0.17dB	29.79 ± 0.18cC	35.23 ± 0.09cC	34.48 ± 0.18D	31.85 ± 0.13bD	42.74 ± 0.06dE	36.59 ± 0.26cE	40.73 ± 0.18aF	17.85 ± 0.13cF
60	94.31 ± 0.06dA	100.0 ± 0.00eA	73.54 ± 0.18dB	78.96 ± 0.13eB	43.06 ± 0.60dC	47.85 ± 0.13dC	41.46 ± 0.09eD	34.52 ± 0.13cD	51.67 ± 0.18eE	55.19 ± 0.13dE	45.87 ± 0.06aF	24.52 ± 0.13dF
70	100.0 ± 0.00eA	100.0 ± 0.0fA	81.91 ± 0.24eB	91.04 ± 0.13fB	50.56 ± 0.06eC	50.59 ± 0.06eC	49.27 ± 0.09fD	46.15 ± 0.06dD	63.33 ± 0.09fE	65.00 ± 0.11eE	51.11 ± 0.12aF	28.96 ± 0.13eF
80	100.0 ± 0.00fA	100.0 ± 0.0gA	88.61 ± 0.12fB	100.0 ± 0.00gA	58.37 ± 0.06fC	56.74 ± 0.13fB	56.35 ± 0.18D	51.19 ± 0.13eC	74.27 ± 0.09gE	73.41 ± 0.13fD	59.48 ± 0.18bF	32.62 ± 0.04fE
NO	20.52 ± 0.18gA	5.63 ± 0.13hA	20.52 ± 0.36gA	5.63 ± 0.13hA	20.52 ± 0.36A	5.63 ± 0.13gA	20.52 ± 0.36A	5.63 ± 0.13fA	20.52 ± 0.36aA	5.63 ± 0.13gA	20.52 ± 0.36cA	5.63 ± 0.13gA
AZ	76.67 ± 0.36hA	75.63 ± 0.13iA	76.67 ± 0.16hA	75.63 ± 0.17iA	76.67 ± 0.16A	75.63 ± 0.17fA	76.67 ± 0.16iA	75.63 ± 0.17gA	76.67 ± 0.16hA	75.63 ± 0.17hA	76.67 ± 0.16dA	75.63 ± 0.17hA
DMSO	0.00iA	0.00jA	0.00iA	0.00jA	0.00iA	0.00gA	0.00jA	0.00hA	0.00iA	0.00iA	0.00eA	0.00iA

Values are mean ± SD; *n* = 3. AG, *A. graveolens*; FA, *F. assa-foetida*; JA, *J. adhatoda*; MU, *M. uniflorum*; NA, *N. arbor-tristis*; VN, *V. negundo*; EO, essential oil, DAT, days after treatment; DMSO, dimethyl sulfoxide; SD, standard deviation; NO, neem oil; AZ, Azoxystrobin. Uppercase letters within a row indicate significant differences among plant EOs at the same dose at different time durations (3 DAT & 5 DAT), while lowercase letters within a column indicate significant differences among doses within the same EO according to Bonferroni's multiple comparison test (*p* < 0.05).

**Table 5:** Summary of comparison of the effects of different plant essential oils (*A. graveolens, F. assa-foetida, J. adhatoda, M. uniflorum, N. arbor-tristis*, and *V. negundo*) at various time durations on the mycelial growth inhibition of *C. gloeosporioides*, tested by using two-way ANOVA followed by Bonferroni's multiple comparisons.

Time duration	Source of interaction	SS	Df	MS	*F*_(DFn, DFd)_	*p-value*
3 DAT	Dose	52,881	9	5,876	*F*_(9, 120)_ = 1,180	*p* < 0.0001
	Plants	21,327	5	4,265	*F*_(5, 120)_ = 857	*p* < 0.0001
	Dose × Plant	11,291	45	251	*F*_(45, 120)_ = 50.4	*p* < 0.0001
5 DAT	Dose	64,141	9	7,127	*F*_(9, 120)_ = 2,271	*p* < 0.0001
	Plants	28,097	5	5,619	*F*_(5, 120)_ = 1,791	*p* < 0.0001
	Dose × Plant	16,848	45	374	*F*_(45, 120)_ = 119	*p* < 0.0001

DAT, days after treatment; SS, sum of squares; Df, degree of freedom; MS, mean square; F, Fisher value; P, probability.

The two-way ANOVA considered six plant EOs tested across different dose levels (20 – 80 μL mL^−1^), along with standard controls, and reported that the dose, plant essential oil (EO), and their interaction significantly influenced fungal growth inhibition of *C. gloeosporioides* at both 3 and 5 DAT (α = 0.05). At 3 DAT, the dose × EO interaction was highly significant [*F*_(45,120)_ = 50.4, *p* < 0.0001], accounting for 13.1% of the total variation, indicating that the magnitude of dose effects differed among EOs. Dose was the predominant factor, explaining 61.4% of the total variation [*F*_(9,120)_ = 1,180, *p* < 0.0001], while plant EO identity contributed 24.8% of the variation [*F*_(5,120)_ = 857, *p* < 0.0001]. These results demonstrate a strong dose-dependent inhibitory response that varied significantly among the EOs tested (Tables 4, 5; Figure 1E).

Bonferroni's multiple comparisons test, at 3 DAT, revealed a clear, consistent, and dose-dependent superiority of AG-EO over all other essential oils (FA-EO, JA-EO, MU-EO, NA-EO, and VN-EO) across the entire tested concentration range (20–80 μL mL^−1^). At 20 and 30 μL mL^−1^, AG-EO showed significantly higher activity than FA-EO (*t* = 3.36, *p* = 0.0052 and *t* = 3.48, *p* = 0.0035, respectively); while differences at 20 μL mL^−1^ and 30 μL mL^−1^ with JA-EO, MU-EO, NA-EO, and VN-EO were highly significant (*p* < 0.0001) (Tables 4, 5; Figure 1E). From 40 to 80 μL mL^−1^, AG-EO was significantly more effective (*p* < 0.0001) than all other essential oils at each concentration, with increasing mean differences as dose increased. The magnitude of mean differences indicates that the relative efficacy gap widened with increasing concentration, particularly against VN-EO and JA-EO. No significant differences were detected among essential oils, neem, and azoxystrobin (*p* > 0.9999), indicating a uniform fungicidal response across treatments (Tables 4, 5; Figure 1E).

In comparison, the bioassay data at 5 DAT demonstrated that all three sources of variation—plants' EOs, dose, and their interaction—were highly significant (*p* < 0.0001), confirming that mycelial growth inhibition is jointly and strongly influenced by EO type, concentration, and their combined effect. Dose is the dominant determinant [58.6%, *F*_(9, 120)_ = 2,271, *p* < 0.0001] of antifungal efficacy at 5 DAT followed by plant EO type [25.7%, *F*_(9, 120)_ = 1,791, *p* < 0.0001] and their interaction [15.4%, *F*_(45, 120)_ = 119, *p* < 0.0001] indicating the substantial variation in the intrinsic antifungal activity of different EOs. Increasing concentration results in a pronounced, systematic increase in percent growth inhibition across essential oils, indicating a strong dose–response relationship (Tables 4, 5; Figure 1E). Bonferroni's multiple comparison test at 5 DAT demonstrates a strong, consistent, and concentration-dependent superiority of AG-EO over all other essential oils (FA-EO, JA-EO, MU-EO, NA-EO, and VN-EO), closely paralleling but intensifying the trends observed at 3 DAT. At 20 μL mL^−1^, AG-EO exhibited significantly higher inhibition than all other essential oils, with large mean differences ranging from 21.1% to 35.9% (*p* < 0.0001). At 30 and 40 μL mL^−1^, AG-EO remained highly significantly superior (*p* < 0.0001) to all other oils, with increasing effect sizes, particularly against VN_EO (*t* = 28.5 and 35.2, *p* < 0.0001, respectively) and JA-EO (*t* = 22.1 and 25.1, *p* < 0.0001, respectively). At 50 μL mL^−1^ and 60 μL mL^−1^, the magnitude of differences further increased (mean differences up to 68.5%), confirming a dose-responsive enhancement of AG_EO efficacy. At 70 μL mL^−1^, AG-EO continued to outperform all other oils significantly (*p* < 0.0001), although the difference relative to FA-EO (*t* = 12, *p* < 0.0001) narrowed compared with lower doses. At 80 μL mL^−1^, AG-EO and FA-EO did not differ significantly (*p* > 0.9999), indicating that both oils achieved maximum or near-maximum inhibitory activity, whereas AG-EO remained significantly more effective than JA-EO, MU-EO, NA-EO, and VN-EO (*p* < 0.0001). All comparisons with controls (neem, azoxy, DMSO) were not significant. Overall, AG-EO's profile closely matches FA-EO, with pronounced divergence from other oils at mid- to high-concentrations (Tables 4, 5). The multiple-comparison analysis at 5 DAT revealed a clear dose-dependent increase in antifungal activity across all essential oils, with AG-EO exhibiting the earliest and strongest response, followed by FA-EO. Oils such as VN showed delayed and weaker responses, becoming significant only at higher concentrations. These findings corroborate the strong dose × EO interaction observed and highlight substantial interspecific variation in antifungal potency of different plants.

#### 3.2.3 Effects of different phytoextracts on the spore count of *C. gloeosporioides*

A dose-dependent reduction in spore count across all plants except FA and solvents was observed. Spore count reduction increased steadily with doses, with near-complete inhibition observed at higher doses of 60–80 μL mL^−1^ for most extracts, particularly in EO and PE extracts (Tables 6, 7). The aqueous and methanolic extracts showed good but slightly slower and lower inhibition, while EA and TCM extracts were variable. Among the six botanicals, AG consistently achieved near-complete inhibition (≥95%−100%) at concentrations ≥30 μg mL^−1^ across all solvent extracts, often matching or exceeding the commercial standard neem and approaching the synthetic fungicide azoxystrobin. FA also performed strongly, achieving >95% inhibition in EO and PE extracts at concentrations of 30 μL mL^−1^ and above. However, FA also shows a highly unusual response. For several solvents (TCM, EA, ME), the values were negative at lower doses (e.g., −233% for TCM at 20 μL mL^−1^), indicating an increase in spore counts rather than inhibition. This effect diminishes with increasing dose, eventually yielding significant spore reduction (e.g., MeOH extract goes from −145% at 20 μL mL^−1^ to +48% at dose 80 μL mL^−1^). This suggests the extract may contain compounds that were stimulatory at low concentrations but inhibitory at high concentrations (Table 6). JA maintained moderate efficacy across most doses, positioning it as a mid-tier performer. Conversely, MU, NA, and VN exhibited weaker activity, particularly in polar extracts, where their inhibition rates plateaued around 85%−93%. Although EO extracts enhanced their performance (≥95% at higher doses), their efficacy remained significantly lower than that of AG and FA.

**Table 6:** *In vitro* bioefficacy of phytoextracts of different plants in different solvents (aqueous, chloroform, ethyl acetate, methanol, and petroleum ether) on the spore count inhibition of *Colletorichum gloeosporioides* 5 days post treatment (*n* = 3).

Plants	Solvents	AQ		TCM		EA		EO		MeOH		PE	
	Dose (μL mL^−1^)	Spore count 5DAT	PROC	Spore count 5DAT	PROC	Spore count 5DAT	PROC	Spore count 5DAT	PROC	Spore count 5DAT	PROC	Spore count 5DAT	PROC
AG	20	(5.55 ± 0.49) × 10^5^	38.12 aA	(3.63 ± 0.53) × 10^4^	95.96 aA	(1.83 ± 0.18) × 10^4^	97.96 aB	(4.17 ± 1.44) × 103	99.54 aC	(1.92 ± 0.35) × 10^4^	97.86 aD	(2.17 ± 0.29) × 10^4^	97.58 aE
	30	(1.03 ± 0.14) × 10^5^	88.57 aA	(2.58 ± 0.38) × 10^4^	97.12 aA	(1.38 ± 0.18) × 10^4^	98.47 aB	(0.0 ± 0.0) × 10^4^	100 aC	(1.83 ± 0.18) × 10^4^	97.96 aD	(1.92 ± 0.14) × 10^4^	97.86 aE
	40	(4.67 ± 0.58) × 10^4^	94.80 aA	(1.88 ± 0.18) × 10^4^	97.91 aA	(1.25 ± 0.11) × 10^4^	98.61 aB	(0.0 ± 0.0) × 10^4^	100 aC	(1.67 ± 0.58) × 10^4^	98.14 aD	(1.33 ± 0.14) × 10^4^	98.51 aE
	50	(3.88 ± 0.18) × 10^4^	95.68 bA	(2.08 ± 0.14) × 10^4^	97.68 bA	(8.33 ± 0.14) × 103	99.07 bB	(0.0 ± 0.0) × 10^4^	100 bC	(4.17 ± 0.14) × 103	99.54 bD	(9.17 ± 0.14) × 103	98.98 bE
	60	(2.88 ± 0.18) × 10^4^	96.79 cA	(1.88 ± 0.18) × 10^4^	97.91 cA	(3.33 ± 0.14) × 103	99.63 cB	(0.0 ± 0.0) × 10^4^	100 cC	(0.0 ± 0.0) × 10^4^	100 cD	(6.92 ± 0.63) × 103	99.23 cE
	70	(2.50 ± 0.35) × 10^4^	97.21 dA	(1.25 ± 0.00) × 10^4^	98.61 dA	(0.0 ± 0.0) × 10^4^	100.0dB	(0.0 ± 0.0) × 10^4^	100 dC	(0.0 ± 0.0) × 10^4^	100 dD	(7.67 ± 1.01) × 103	99.14 dE
	80	(9.17 ± 0.29) × 103	89.50 aB	(1.10 ± 0.14) × 10^4^	−233.09 aE	(0.0 ± 0.0) × 10^4^	−121.51 aC	(0.0 ± 0.0) × 10^4^	96.65 aA	(0.0 ± 0.0) × 10^4^	−145.49 aD	(5.00 ± 0.00) × 103	91.82 aA
FA	20	(9.42 ± 0.14) × 10^4^	91.45 aB	(2.99 ± 0.16) × 10^6^	−168.96 aE	(1.99 ± 0.09) × 10^6^	−104.65 aC	(3.00 ± 0.25) × 10^4^	100 aA	(2.20 ± 0.25) × 10^6^	−125.28 aD	(7.33 ± 0.52) × 10^4^	100 aA
	30	(7.67 ± 0.38) × 10^4^	92.75 aB	(2.41 ± 0.16) × 10^6^	−120.63 aE	(1.84 ± 0.01) × 10^6^	−90.85 aC	(0.0 ± 0.0) × 10^4^	100 aA	(2.02 ± 0.06) × 10^6^	−96.28 aD	(0.0 ± 0.0) × 10^4^	100 aA
	40	(6.50 ± 0.43) × 10^4^	94.52 aB	(1.98 ± 0.21) × 10^6^	−71.75 aE	(1.71 ± 0.17) × 10^6^	−12.27 aC	(0.0 ± 0.0) × 10^4^	100 aA	(1.76 ± 0.14) × 10^6^	−70.07 aD	(0.0 ± 0.0) × 10^4^	100 aA
	50	(4.92 ± 0.14) × 10^4^	97.77 aB	(1.54 ± 0.11) × 10^6^	−52.79 aE	(1.01 ± 0.12) × 10^6^	9.29 aC	(0.0 ± 0.0) × 10^4^	100 aA	(1.53 ± 0.04) × 10^6^	−25.46 aD	(0.0 ± 0.0) × 10^4^	100 aA
	60	(2.00 ± 0.18) × 10^4^	98.70 aB	(1.37 ± 0.06) × 10^6^	−9.67 aE	(8.13 ± 0.15) × 10^5^	43.49 aC	(0.0 ± 0.0) × 10^4^	100 aA	(1.13 ± 0.11) × 10^6^	20.26 aD	(0.0 ± 0.0) × 10^4^	100 aA
	70	(1.17 ± 0.35) × 10^4^	99.40 bB	(9.83 ± 0.28) × 10^5^	26.39 bE	(5.07 ± 0.17) × 10^5^	74.26 bC	(0.0 ± 0.0) × 10^4^	100 bA	(7.15 ± 0.11) × 10^5^	48.14 bD	(0.0 ± 0.0) × 10^4^	100 bA
	80	(5.42 ± 0.88) × 103	71.28 aB	(6.60 ± 0.29) × 10^5^	63.38 aD	(2.31 ± 0.66) × 10^5^	80.85 aA	(0.0 ± 0.0) × 10^4^	89.68 aC	(4.65 ± 0.49) × 10^5^	83.09 aA	(0.0 ± 0.0) × 10^4^	72.30 aA
JA	20	(2.58 ± 0.21) × 10^5^	91.91 bB	(3.28 ± 0.20) × 10^5^	67.10 bD	(1.72 ± 0.21) × 10^5^	91.64 bA	(9.25 ± 1.09) × 10^4^	91.82 bC	(1.52 ± 0.38) × 10^5^	86.25 bA	(2.48 ± 0.13) × 10^5^	80.02 bA
	30	(7.25 ± 1.06) × 10^4^	91.91 cB	(2.95 ± 0.13) × 10^5^	73.23 cD	(7.50 ± 0.35) × 10^4^	91.82 cA	(7.33 ± 0.29) × 10^4^	94.61 cC	(1.23 ± 0.35) × 10^5^	89.50 cA	(1.79 ± 0.19) × 10^5^	87.92 cA
	40	(7.25 ± 1.06) × 10^4^	93.68 dB	(2.40 ± 0.43) × 10^5^	82.43 dD	(7.33 ± 0.50) × 10^4^	92.47 dA	(4.83 ± 0.38) × 10^4^	96.19 dC	(9.42 ± 0.63) × 10^4^	91.36 dA	(1.08 ± 0.14) × 10^5^	88.94 dA
	50	(5.67 ± 0.63) × 10^4^	94.61 eB	(1.58 ± 0.18) × 10^5^	84.76 eD	(6.75 ± 0.87) × 10^4^	93.40 eA	(3.42 ± 0.14) × 10^4^	98.23 eC	(7.75 ± 0.35) × 10^4^	93.96 eA	(9.92 ± 0.38) × 10^4^	91.36 eA
	60	(4.83 ± 0.52) × 10^4^	95.54 fB	(1.37 ± 0.13) × 10^5^	86.25 fD	(5.92 ± 0.14) × 10^4^	94.61 fA	(1.58 ± 0.14) × 10^4^	100 fC	(5.42 ± 0.35) × 10^4^	98.51 fA	(7.75 ± 0.66) × 10^4^	93.03 fA
	70	(4.00 ± 0.50) × 10^4^	97.96 gB	(1.23 ± 0.38) × 10^5^	90.06 gD	(4.83 ± 0.38) × 10^4^	96.28 gA	(0.0 ± 0.0) × 10^4^	100 gC	(1.33 ± 0.14) × 10^4^	99.41 gA	(6.25 ± 0.25) × 10^4^	94.42 gA
	80	(1.83 ± 0.26) × 10^4^	82.99 aB	(8.92 ± 0.38) × 10^4^	92.29 aB	(3.33 ± 0.38) × 10^4^	92.09 aB	(0.0 ± 0.0) × 10^4^	98.70 aD	(5.33 ± 0.15) × 103	85.59 aA	(5.00 ± 0.66) × 10^4^	91.73 aC
MU	20	(1.53 ± 0.10) × 10^5^	83.53 aB	(6.92 ± 0.38) × 10^4^	92.66 aB	(7.10 ± 1.07) × 10^4^	94.14 aB	(1.17 ± 0.14) × 10^4^	98.03 aD	(1.29 ± 0.12) × 10^5^	86.28 aA	(7.42 ± 0.38) × 10^4^	92.38 aC
	30	(1.48 ± 0.87) × 10^5^	84.57 aB	(6.58 ± 0.80) × 10^4^	92.94 aB	(5.25 ± 0.32) × 10^4^	93.68 aB	(1.77 ± 0.03) × 10^4^	98.14 aD	(1.23 ± 0.75) × 10^5^	86.34 aA	(6.83 ± 0.38) × 10^4^	92.66 aC
	40	(1.38 ± 0.38) × 10^5^	85.41 aB	(6.33 ± 0.52) × 10^4^	93.12 aB	(5.67 ± 0.24) × 10^4^	93.45 aB	(1.67 ± 0.14) × 10^4^	98.23 aD	(1.23 ± 0.43) × 10^5^	86.43 aA	(6.58 ± 0.76) × 10^4^	93.40 aC
	50	(1.31 ± 0.58) × 10^5^	85.87 aB	(6.17 ± 0.52) × 10^4^	93.68 aB	(5.88 ± 0.12) × 10^4^	93.36 aB	(1.58 ± 0.14) × 10^4^	98.42 aD	(1.22 ± 0.80) × 10^5^	86.80 aA	(5.92 ± 0.14) × 10^4^	94.14 aC
	60	(1.27 ± 0.38) × 10^5^	88.38 aB	(5.67 ± 0.52) × 10^4^	95.82 aB	(5.96 ± 0.06) × 10^4^	93.40 aB	(1.42 ± 0.14) × 10^4^	98.79 aD	(1.18 ± 0.38) × 10^5^	90.71 aA	(5.25 ± 0.50) × 10^4^	94.61 aC
	70	(1.04 ± 0.12) × 10^5^	90.06 bB	(3.75 ± 0.50) × 10^4^	96.93 bB	(5.92 ± 1.53) × 10^4^	93.68 bB	(1.08 ± 0.14) × 10^4^	99.16 bD	(8.33 ± 0.58) × 10^4^	93.12 bA	(4.83 ± 0.63) × 10^4^	96.10 bC
	80	(8.92 ± 0.38) × 10^4^	86.90 aB	(2.75 ± 0.25) × 10^4^	94.80 aB	(5.67 ± 0.33) × 10^4^	94.52 aB	(7.50 ± 0.25) × 103	96.00 aD	(6.17 ± 1.42) × 10^4^	90.43 aA	(3.50 ± 0.25) × 10^4^	96.38 aC
NA	20	(1.18 ± 0.08) × 10^5^	87.27 aB	(4.67 ± 0.63) × 10^4^	95.63 aB	(4.92 ± 0.38) × 10^4^	96.00 aB	(3.58 ± 0.14) × 10^4^	98.23 aD	(8.58 ± 1.01) × 10^4^	91.17 aA	(3.25 ± 0.43) × 10^4^	96.38 aC
	30	(1.14 ± 0.14) × 10^5^	89.13 aB	(3.92 ± 0.52) × 10^4^	96.00 aB	(3.58 ± 0.29) × 10^4^	96.47 aB	(1.58 ± 0.14) × 10^4^	99.44 aD	(7.92 ± 0.80) × 10^4^	91.64 aA	(3.25 ± 0.43) × 10^4^	96.75 aC
	40	(9.75 ± 1.09) × 10^4^	89.68 aB	(3.58 ± 0.38) × 10^4^	96.65 aB	(3.17 ± 0.29) × 10^4^	96.75 aB	(5.00 ± 0.00) × 103	99.44 aD	(7.50 ± 0.66) × 10^4^	92.10 aA	(2.92 ± 0.38) × 10^4^	97.63 aC
	50	(9.25 ± 0.66) × 10^4^	91.26 aB	(3.00 ± 0.43) × 10^4^	96.75 aB	(2.92 ± 0.14) × 10^4^	96.84 aB	(5.00 ± 0.00) × 103	99.44 aD	(7.08 ± 0.53) × 10^4^	92.84 aA	(2.13 ± 0.18) × 10^4^	97.78 aC
	60	(7.83 ± 1.04) × 10^4^	92.38 bB	(2.92 ± 0.38) × 10^4^	97.21 bB	(2.83 ± 0.38) × 10^4^	97.12 bB	(5.00 ± 0.00) × 103	99.44 bD	(6.42 ± 0.76) × 10^4^	92.47 bA	(1.99 ± 0.26) × 10^4^	98.33 bC
	70	(6.83 ± 0.76) × 10^4^	94.24 bB	(2.50 ± 0.25) × 10^4^	98.14 bB	(2.58 ± 0.14) × 10^4^	97.30 bB	(5.00 ± 0.00) × 103	100 bD	(6.75 ± 0.87) × 10^4^	94.61 bA	(1.50 ± 0.14) × 10^4^	98.51 bC
	80	(5.17 ± 0.14) × 10^4^	88.75 aB	(1.67 ± 0.14) × 10^4^	89.59 aB	(2.42 ± 0.14) × 10^4^	90.33 aB	(0.0 ± 0.0) × 10^4^	90.89 aD	(4.83 ± 0.38) × 10^4^	90.99 aA	(1.33 ± 0.14) × 10^4^	90.89 aC
VN	20	(1.01 ± 0.06) × 10^5^	89.03 aB	(9.33 ± 0.29) × 10^4^	90.89 aB	(8.67 ± 0.14) × 10^4^	91.17 aB	(8.17 ± 0.63) × 10^4^	91.64 aD	(8.08 ± 0.14) × 10^4^	91.26 aA	(8.17 ± 0.38) × 10^4^	91.26 aC
	30	(9.83 ± 0.29) × 10^4^	89.50 aB	(8.17 ± 0.14) × 10^4^	91.08 aB	(7.92 ± 0.52) × 10^4^	91.73 aB	(7.50 ± 0.90) × 10^4^	92.29 aD	(7.83 ± 0.38) × 10^4^	91.45 aA	(7.83 ± 0.38) × 10^4^	92.01 aC
	40	(9.42 ± 0.38) × 10^4^	90.24 aB	(8.00 ± 0.43) × 10^4^	91.26 aB	(7.42 ± 0.72) × 10^4^	92.38 aB	(6.92 ± 0.52) × 10^4^	92.53 aD	(7.67 ± 0.38) × 10^4^	91.64 aA	(7.17 ± 0.38) × 10^4^	92.94 aC
	50	(8.75 ± 0.25) × 10^4^	91.45 aB	(7.83 ± 0.38) × 10^4^	92.47 aB	(6.83 ± 0.38) × 10^4^	93.22 aB	(6.70 ± 0.36) × 10^4^	92.83 aD	(7.50 ± 0.50) × 10^4^	92.38 aA	(6.33 ± 0.29) × 10^4^	93.49 aC
	60	(7.67 ± 0.29) × 10^4^	92.84 bB	(6.75 ± 0.25) × 10^4^	92.66 bB	(6.08 ± 0.29) × 10^4^	93.77 bB	(6.43 ± 0.51) × 10^4^	93.05 bD	(6.83 ± 0.14) × 10^4^	93.22 bA	(5.83 ± 0.58) × 10^4^	94.89 bC
	70	(6.42 ± 0.38) × 10^4^	94.33 bB	(6.58 ± 0.14) × 10^4^	94.05 bB	(5.58 ± 0.14) × 10^4^	94.61 bB	(6.23 ± 0.42) × 10^4^	93.16 bD	(6.08 ± 0.14) × 10^4^	93.49 bA	(4.58 ± 0.14) × 10^4^	95.54 bC
	80	(5.08 ± 0.14) × 10^4^	94.33 bB	(5.33 ± 0.14) × 10^4^	94.05 bB	(4.83 ± 0.14) × 10^4^	94.61 bB	(6.13 ± 0.32) × 10^4^	93.16 bD	(5.83 ± 0.38) × 10^4^	93.49 bA	(4.00 ± 0.25) × 10^4^	95.54 bC
AZ		(0.0 ± 0.0) × 10^4^	100.00	(0.0 ± 0.0) × 10^4^	100.00	(0.0 ± 0.0) × 10^4^	100.00	(0.0 ± 0.0) × 10^4^	100.00	(0.0 ± 0.0) × 10^4^	100.00	(0.0 ± 0.0) × 10^4^	100.00
DMSO		(8.97 ± 1.45) × 10^5^	0.00	(8.97 ± 1.45) × 10^5^	0	(8.97 ± 1.45) × 10^5^	0.00	(8.97 ± 1.45) × 10^5^	0.00	(8.97 ± 1.45) × 10^5^	0.00	(8.97 ± 1.45) × 10^5^	0.00

AG, *A. graveolens*; AZ, Azoxystrobin; FA, *F. assa-foetida*; JA, *J. adhatoda*; MU, *M. uniflorum*; NA, *N. arbor-tristis*; NO, neem oil; VN, *V. negundo*; EO, essential oil; DAT, days after treatment; DMSO, dimethyl sulfoxide; SD, standard deviation; AQ, aqueous extract; TCM, chloroform extract; EA, ethyl acetate extract; ME, methanol extract; PE, petroleum ether extract; DAT, days after treatment; PROC, percent reduction over control. Uppercase letters within a row indicate significant differences among different plants at the same dose, whereas lowercase letters within a column indicate significant differences among doses within the solvents according to Bonferroni's multiple comparison test (*p* < 0.05).

**Table 7:** Summary of comparison of the effects of different plants (*A. graveolens, F. assa-foetida, J. adhatoda, M. uniflorum, N. arbor-tristis*, and *V. negundo*) extracted in different solvents (Aqueous, Chloroform, Ethyl acetate, Methanol, and Petroleum ether) on the spore count inhibition of *C. gloeosporioides* tested by using two-way ANOVA followed by Bonferroni's multiple comparisons (*p* < 0.05).

Plant	Source of interaction	SS	DF	MS	*F*_(DFn, DFd)_	*p-value*
AG	Dose	12,233,150	9	1,359,239	*F*_(9, 120)_ = 79.5	*p* < 0.0001
	Solvent	594,700	5	118,940	*F*_(5, 120)_ = 6.95	*p* < 0.0001
	Interaction	841,746	45	18,705	*F*_(45, 120)_ = 1.09	*p* = 0.3443
FA	Dose	14,638,589	9	1,626,510	*F*_(9, 120)_ = 30.4	*p* < 0.0001
	Solvent	25,586,271	5	5,117,254	*F*_(5, 120)_ = 95.6	*p* < 0.0001
	Interaction	13,081,588	45	290,702	*F*_(45, 120)_ = 5.43	*p* < 0.0001
JA	Dose	10,309,656	9	1,145,517	*F*_(9, 120)_= 58.1	*p* < 0.0001
	Solvent	615,825	5	123,165	*F*_(5, 120)_= 6.25	*p* < 0.0001
	Interaction	401,593	45	8,924	*F*_(45, 120)_ = 0.453	*p* = 0.9984
MU	Dose	9,592,020	9	1,065,780	*F*_(9, 120)_ = 49.1	*p* < 0.0001
	Solvent	525,113	5	105,023	*F*_(5, 120)_= 4.84	*p* = 0.0005
	Interaction	250,802	45	5,573	*F*_(45, 120)_ = 0.257	*P* > 0.9999
NA	Dose	10,413,620	9	1,157,069	*F*_(9, 120)_ = 64.4	*p* < 0.0001
	Solvent	430,618	5	86,124	*F*_(5, 120)_ = 4.80	*p* = 0.0005
	Interaction	211,754	45	4,706	*F*_(45, 120)_ = 0.262	*p* > 0.9999
VN	Dose	10,431,996	9	1,159,111	*F*_(9, 120)_= 66.7	*p* < 0.0001
	Solvent	398,937	5	79,787	*F*_(5, 120)_ = 4.59	*p* = 0.0007
	Interaction	197,578	45	4,391	*F*_(45, 120)_ = 0.253	*p* > 0.9999

AG, *A. graveolens*; FA, *F. assa-foetida*;, JA, *J. adhatoda*; MU, *M. uniflorum*; NA, *N. arbor-tristis*; VN, *V. negundo*; EO, essential oil; DAT, days after treatment; DMSO, dimethyl sulfoxide; SD, standard deviation; AQ, aqueous extract; TCM, chloroform extract; EA, ethyl acetate extract; ME, methanol extract; PE, petroleum ether extract; DAT, days after treatment; SS, sum of squares; Df, degree of freedom; MS, mean square; F, fisher value; P, probability.

Two-way ANOVA across all six plant extracts demonstrated that dose was the dominant factor influencing antifungal activity, consistently accounting for the majority of the variation in spore percent reduction (85%−95%, *p* < 0.0001) (Tables 6, 7). Solvent type also had a significant but secondary effect (2%−7% of variation, *p* < 0.0001), indicating that the extraction medium modulated the magnitude of the antifungal response. In the case of AG, two-way ANOVA revealed that dose had a highly significant effect on spore count [*F*_(9,120)_ = 79.5, *p* < 0.0001], accounting for 77.8% of total variation, whereas solvent effects were significant but comparatively minor [*F*_(5,120)_ = 6.95, *p* < 0.0001]. No significant dose × solvent interaction was observed, indicating a consistent dose response across solvents. Bonferroni's *post hoc* analysis showed a pronounced dose-dependent reduction in spore counts for aqueous extracts (*p* < 0.0001), whereas organic solvent extracts exhibited strong antifungal activity even at lower doses [*t* = 5.19, *p* < 0.0001 (TCM); *t* = 5.71, *p* < 0.0001 (EA); *t* = 6.37, *p* < 0.0001 (EO); *t* = 5.68, *p* < 0.0001 (MeOH), and t = 5.60, *p* < 0.0001 (PE)], with limited additional gains at higher concentrations. At moderate to high doses, differences among solvents were largely not significant (*p* > 0.999), suggesting convergence of efficacy. All extracts differed significantly from the DMSO control, confirming the genuine antifungal activity of AG extracts against spore production (Tables 6, 7).

In the case of FA, solvent type, dose, and their interaction had a highly significant effect on spore count (solvent: *p* < 0.0001, 42.8% of variation; dose: *p* < 0.0001, 24.5% of variation; interaction: *p* < 0.0001, 21.9% variation). *Post hoc* analysis indicated that extracts prepared with chloroform, methanol, and ethyl acetate (*p* < 0.0001) resulted in significantly higher spore counts compared to the aqueous extract (*p* < 0.001) (Tables 6, 7). Negative inhibition values, i.e., an increase in spores at lower doses (e.g., FA in EA, TCM, and MeOH extracts), suggest possible fungal stimulation. Conversely, extracts prepared with EO (*t* = 0.71, *p* > 0.9999) or petroleum ether (*t* = 0.19, *p* > 0.9999) did not differ significantly from the aqueous extracts. The spore count reduction in JA was significantly affected by solvent type (solvent: *p* < 0.0001, 4.5% of variation) and dose had a highly significant effect (*p* < 0.0001, 75.3% of variation). Within JA-AQ, except at 80 μL mL^−1^ (*p* = 0.0136), the dose-response in the majority of doses (30–70 μL mL^−1^) was not significant (*p* = 0.3583 to 0.075), suggesting a threshold effect at higher concentrations. Among the different extracts, Bonferroni's multiple comparisons test revealed no significant differences (*p* > 0.05) between the aqueous extract (JA-AQ) and other solvent extracts at any tested dose (20–80). Similarly, JA-AQ did not differ significantly from neem, azoxystrobin, or the solvent control, indicating comparable efficacy across extracts (Tables 6, 7). A highly significant effect of dose on the spore counts of MU [*F*_(_9, _120)_ = 49.1, *p* < 0.0001], explaining 73.9% of the total variation, and a significant effect of solvent was also observed [*F*_(_5, _120)_ = 4.84, *p* = 0.0005], accounting for 4.05% of the variation. Dose-wise comparisons revealed no significant differences between dose 20 μL mL^−1^ and higher concentrations (30–80 μL mL^−1^) for any MU extract, indicating the absence of a dose-dependent response. Solvent-wise comparisons at each dose similarly showed no significant differences among aqueous, organic, and essential oil extracts. All MU extracts performed significantly better than the solvent control (DMSO) but were inferior to the chemical standard (azoxystrobin) and statistically comparable to neem (Tables 6, 7). Similar results were observed for NA, and VN revealed a dose-dependent reduction in spore counts across all plant extracts, with dose contributing the highest variation (>78%) in each case (*p* < 0.0001). Among the solvents tested, solvent type also significantly influenced antifungal efficacy (variation: NA = 3.26%, VN = 3.04%; all *p* < 0.0001), highlighting the critical role of solvent polarity in extracting bioactive antifungal compounds. Across species, EO consistently demonstrated the strongest inhibitory activity (>98% spore reduction), followed by PE extracts, whereas AQ and MeOH fractions showed comparatively lower activity. Bonferroni's *post hoc* analysis revealed no significant dose-dependent differences among NA and VN extracts across concentrations of 20–80 μL mL^−1^. Solvent-wise comparisons at each dose also showed no significant variation among aqueous, organic, and essential oil extracts. All NA and VN extracts were significantly superior to the solvent control (DMSO), were statistically comparable to neem, and inferior to the chemical standard azoxystrobin (Tables 6, 7). Overall, the results highlight that dose is the dominant factor influencing spore reduction, while solvent choice also significantly modulates spore inhibition, with nonpolar extracts (EO, PE) generally outperforming polar ones (EA, TCM, MeOH, AQ).

#### 3.2.4 Minimum inhibitory concentration

The MIC_50_ values for the effective botanicals, determined by the microdilution method, indicated ≥50%. The MIC_50_ values for effective botanicals ranged from 14.1 to 75.3 μL mL^−1^ (Table 8). The effective MIC_50_ values for AG ranged from 14.1 ± 3.82 to 53.65 ± 9.28 μL mL^−1^, with the least value found in AG-EO. It was followed by the FA phytoextracts, i.e., between 31.50 ± 6.97 and 57.60 ± 7.85 μL mL^−1^. According to the radial growth inhibition (%), AG-EO and its MeOH phytoextracts were the most effective, with more than 50% inhibition against *C. gloeosporioides* at 20 and 50 μL mL^−1^, respectively. Thus, the MIC values were 14.10 ± 3.82 and 43.80 ± 12.94 μL mL^−1^ in both of the treatments, respectively. Besides, the MIC_50_ value of AG-MeOH phytoextract was observed to be higher than that of FA-EO and FA-PE extract (Table 8). Based on the concentrations, FA-EO and its petroleum ether-derived phytoextracts were more effective than AG-EO at lower doses. NA-EO had the lower MIC_50_ value of 58.00 ± 6.23 μL mL^−1^ among the AG and FA phytoextracts. Comparatively, the MIC_50_ values of JA-MeOH and MU-EO were 67.5 ± 8.84 μL mL^−1^ and 75.30 ± 9.32 μL mL^−1^, respectively. The MIC_90_ was observed to be more than 70 μL mL^−1^ in each phytoextract treatment except AG-EO, which exhibited efficient antifungal activity with MIC_90_ as 32.0 ± 6.48 μL mL^−1^ (Table 8). The lower MIC of AG-EO showed that the phytocompounds and volatiles in AG-EO were much more potent against the fungal proliferation. AG-MeOH showed a better inhibitory effect than its extraction obtained from moderately polar solvent ethyl acetate (AG-EA). The phytocompounds in AG were overall effective against *C. gloeosporioides* as compared to other phytoextracts, indicating their potential inhibitory interactions with the fungal cells.

**Table 8:** Minimum inhibitory concentration (MIC) of phytoextracts and essential oils against *Colletorichum gloeosporioides* as determined by agar poisoned plate assay.

Phytoextracts	MIC_50_ (μL mL^−1^)	MIC_90_ (μL mL^−1^)
*A. graveolens* (EA)	53.65 ± 9.28	81.20 ± 6.55
*A. graveolens* (EO)	14.10 ± 3.82	32.00 ± 6.48
*A. graveolens* (MeOH)	43.80 ± 12.94	82.00 ± 10.41
*F. assa-foetida* (AQ)	57.60 ± 7.85	77.75 ± 6.21
*F. assa-foetida* (EO)	38.50 ± 8.06	70.70 ± 6.60
*F. assa-foetida* (PE)	31.50 ± 6.97	70.50 ± 4.93
*J. adhatoda* (MeOH)	67.50 ± 8.85	80.00 ± 8.12
*M. uniflorum* (EO)	75.30 ± 9.32	85.00 ± 9.67
*N. arbor-tristis* (EO)	58.00 ± 6.23	85.00 ± 6.58

AQ, aqueous extract; TCM, chloroform extract; EA, ethyl acetate extract; MeOH, methanol extract; PE, petroleum ether extract; EO, essential oil.

#### 3.2.5 Ultrastructural morphological characteristics of *C. gloeosporioides*

**Figure 4:**
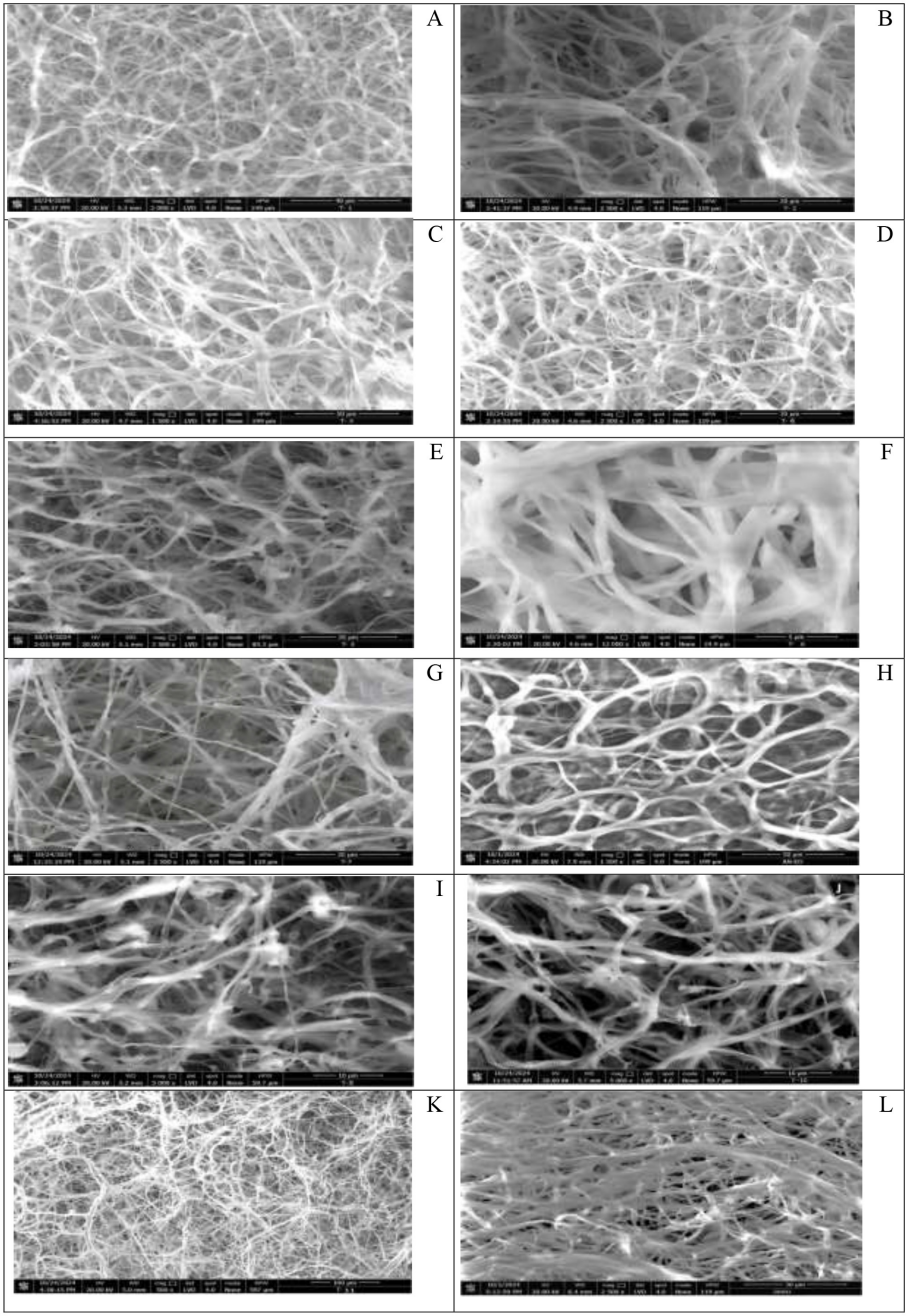
SEM images of the effect of different phytoextracts and essential oils on the mycelial ultra-morphology of *Colletotrichum gloeosporioides*. **(A)** FA-PE, 40 μL/mL; **(B)** FA-AQ, 60 μL/mL; **(C)** AG-MeOH, 50 μL/mL; **(D)** JA- MeOH, 70 μL/mL; **(E)** FA-EO, 40 μL/mL; **(F)** MU-EO, 70 μL/mL; **(G)** AG-EA, 60 μL/mL; **(H)** AG-EO 20 μL/mL; **(I)** NA-EO 60 μL/mL; **(J)** Neem; **(K)** Azoxystrobin; **(L)** control-DMSO. *Anethum graveolens* (AG), *Ferula assa-foetida* (FA), *Justicia adhatoda* (JA), *Macrotyloma uniflorum* (MU), *Nyctanthes arbor-tristis* (NA), *Vitex negundo* (VN); AQ, Aqueous; EA, ethyl acetate; EO, essential oil; TCM, chloroform; MeOH, methanol; PE, petroleum ether.

The hyphal growth of *C. gloeosporioides* treated with different phytoextracts was scanned and imaged with SEM. The morphological characteristics of the fungal mycelium were observed for each treatment and control. It was found that the phytoextracts and EOs changed the morphological characteristics of *C. gloeosporioides*. The mycelium was found to be distorted and wrinkled in each phytoextract treatment as compared to the control (DMSO) (Figure 4). The mycelial arrangement in control (DMSO) was densely compact, homogeneous, and interconnected, whereas the treated culture plates showed either loose, disconnected, or shriveled branches. The hyphae in control appeared smooth and uniform, and the fungal colony was found to have a thicker mat-like appearance (Figure 4L). It was followed by the botanical control, neem (Figure 4J), which showed shriveled fungal growth but less than phytoextract-treated cultures. The hyphal arrangement was similar to that in DMSO. Conversely, each treatment on the culture plate caused damage to mycelial layers and cytoplasmic depletion of the fungal hyphae. The culture treated with AG-EO (Figure 4H) showed a highly desiccated, loosely compact mat, followed by the culture treated with AG-MeOH (Figure 4C). The colony observed in culture treated with FA-PE (Figure 4A) was abundant and interconnected. However, the mycelial branches were very thin and withered, whereas the fungal hyphae in culture treated with AG-EA (Figure 4G) were more twisted and distorted in structure. The hyphal morphology of the culture treated with FA-EO was also interconnected, as in the FA-PE treatment, but exhibited a more irregular and twisted structure (Figures 4A, E). Conversely, the FA-AQ-treated culture showed a denser, smoother fungal mat, but also exhibited irregularities and bulging hyphae (Figure 4B). It was also found that in the culture treated with NA-EO (Figure 4I), the mycelial mat was interconnected, but the hyphal branches were irregularly spread and overly bulged. Similarly, the mycelial mat of the fungal colony treated with JA-MeOH was roughly proliferated with wilted and twisted branches (Figure 4D). Comparatively, the colony treated with MU-EO also showed shriveled hyphae, but the fungal mat appeared slightly denser and smoother (Figure 4F). Furthermore, the positive chemical control, azoxystrobin, exhibited the greatest inhibition of mycelial growth. The mycelia were reduced to a wrinkled structure under chemical control, which overall appeared as a thinner fungal mat of *C. gloeosporioides* compared to other treatments (Figure 4K).

#### 3.2.6 Phytochemical analysis

Based on the initial antifungal assay, GC–MS was employed to identify the active compounds of the most effective treatments, i.e., EOs (AG, FA, JA, MU, NA) and phytoextracts (AG EA and MeOH extracts; FA-AQ and PE; JA-MeOH extract). A total of 137 compounds were identified from the 10 most effective treatments. The composition of their total active compounds ranged between 84.6% and 97.65%. The most abundant 10 active compounds from each phytoextract treatment, along with their retention times (RTs), and percent composition, were presented in Table 9. A total of 28 bioactive compounds were identified from the FA-PE, followed by NA*-*EO (27 compounds). Fatty acids represented the most abundant category, accounting for 25.87% of the total composition, followed by alcohols (13.48%) and epoxides (10.58%). Sulfur-containing compounds comprised 7.11 %, while esters (6.92%), terpenoids (6.66%), and terpenes (5.79%) also formed significant proportions. Hydrocarbons and vitamins each contributed 5.03%, while ketones (4.28%) and ethers (3.37%) occurred in moderate amounts. Aromatics (3.21%), alkynes (2.99%), alkaloids (1.86%), steroids (2.99%), and acetals (1.05%) were present in lower proportions. The distribution pattern suggests a predominance of lipid-derived and terpenoid-based constituents, supported by the presence of functionally diverse secondary metabolites. The major components in AG-EO consisted of Tricyclo[4.4.0.02,8]dec-3-en-5-ol (23.11%) and 1-ethyl-3-methoxybenzene (18.59%); 4,7-dimethoxy-5-prop-1-enyl-1,3-benzodioxole (30.08% and 26.57% in AG-EA and MeOH extract, respectively) (Table 9). The FA*-*EO and AQ consisted of (Z)-octadec-9-enoic acid at a concentration of 36.81% each, while FA-PE reported 14.35% methyl(6Z,9Z,12Z)-hexadeca-6,9,12,15-tetraenoate. The major active constituent of JA was (E)-octadec-13-enoic acid (50.76% in EO and 33.33% in MeOH extract) (Table 9). Similar to FA-EO, the MU-EO also consisted of (Z)-octadec-9-enoic acid (33.34%) as the major phytoconstituent, followed by 1,3-dihydroxypropan-2-yl octadecanoate (26.37%) while 5-methyl-1-oxido-2,1,3-benzoxadiazol-1-ium (9.98%), 3-(3-hydroxybutyl)-2,4,4-trimethylcyclohex-2-en-1-one (9.01%), and (2E)-2-benzylideneoctanal (7.78 %) were the main active compounds in the NA-EO (Table 9). Apart from these major components, several bioactive compounds like α-pinene, ginnone, limonene, trans-dihydrocarvone, α-farnesene, erucic acid, ocimene, ambrosin, cis-raphasatin, β-thujene, squalene, phytol, lupeol, stigmasterol, n-hexadecanoic acid, eugenol, cinnamyl cinnamate, etc. were also reported in minor quantities.

**Table 9:** Chemical composition and relative abundance % of the top five bioactive compounds of the most effective essential oils and phytoextracts using GC-MS.

Plant	Essential oil			Ethyl acetate extract			Methanol extract		
	IUPAC name	RT	% Composition	IUPAC name	RT	% Composition	IUPAC name	RT	% Composition
*Anethum graveolens*	Tricyclo[4.4.0.02,8]dec-3-en-5-ol	15.65	23.11	4,7-dimethoxy-5-prop-1-enyl-1,3-benzodioxole	27.77	30.08	4,7-dimethoxy-5-prop-1-enyl-1,3-benzodioxole	27.74	26.57
	1-ethyl-3-methoxybenzene	9.36	18.59	(Z)-octadec-9-enoic acid	36.82	17.46	(Z)-octadec-9-enoic acid	36.83	23.70
	7-methylidenebicyclo [3.3.1]nonan-3-one	15.80	17.47	(2E)-2-(3,3-dimethylcyclohexylidene)acetaldehyde	14.41	14.94	5-methyl-4-methylidene-2,3,3a,5,6,6a-hexahydropentalen-1-one	15.53	13.50
	(Z)-octadec-11-enoic acid	36.89	12.99	5-methyl-4-methylidene-2,3,3a,5,6,6a-hexahydropentalen-1-one	15.54	14.50	(2E)-2-(3,3dimethylcyclohexylidene) acetaldehyde	14.38	11.75
	oxiran-2-ylmethyl (Z)-octadec-9-enoate	40.69	4.78	6-methylidene-1,2,3,3a,4,5,7,7a-octahydroindene	9.31	10.17	4-methyl-1-prop-1-en-2-ylcyclohexene	9.28	9.01
	(9E,12E)-octadeca-9,12-dienoic acid	37.39	3.44	1,3-dihydroxypropan-2-yl octadecanoate	40.69	4.78	1,3-dihydroxypropan-2-yl octadecanoate	40.69	6.16
	Octadecanoic acid	37.11	3.22	11-pentan-3-ylhenicosane	49.33	1.79	(2R,5R)-2-methyl-5-prop-1-en-2-ylcyclohexan-1-one	14.19	4.19
	Hexadecanoic acid	34.33	2.18	(2R,5R)-2-methyl-5-prop-1-en-2-ylcyclohexan-1-one	14.20	1.52	Octadec-17-ynoic acid	38.03	1.05
	methyl 8-[2-[[2-[(2-ethylcyclopropyl)methyl]cyclopropyl]methyl]cyclopropyl]octanoate	40.62	2.03	nonacosan-10-one	49.05	1.28	Hexadecanoic acid	34.33	0.91
	(1R,5S)-3,6,6-trimethylbicyclo [3.1.1] hept-2-ene	8.11	1.62	Octadec-17-ynoic acid	38.05	0.82	Oxiran-2-ylmethyl hexadecanoate	40.98	0.76
*Ferula assa-foetida*	(Z)-octadec-9-enoic acid	36.81	34.78	(Z)-octadec-9-enoic acid	36.81	22.36	methyl (6Z,9Z,12Z)-hexadeca-6,9,12,15-tetraenoate	21.03	14.35
	2-[[(Z)-prop-1-enyl]disulfanyl]butane	13.41	12.75	1-(methyldisulfanyl)-1-methylsulfanylpropane	20.75	14.27	2-[[(Z)-prop-1-enyl]disulfanyl]butane	13.51	9.07
	1,3-dihydroxypropan-2-yl octadecanoate	40.69	10.88	6-(3-hydroxyprop-1-en-2-yl)-4,8a-dimethyl-1,3,5,6,7,8-hexahydronaphthalen-2-one	33.64	12.95	4-methyl-5-(5-methyl-2-phenyl-1,3-dioxolan-4-yl)-2-phenyl-1,3-dioxolane	13.37	8.97
	methyl 2-methoxy-3-methylbutanoate	27.08	8.77	oxiran-2-ylmethyl (Z)-octadec-9-enoate	40.67	10.49	ethyl 2-oxo-3-sulfanylpropanoate	21.18	5.95
	Hexadecanoic acid	34.33	3.63	2-[[(Z)-prop-1-enyl]disulfanyl]butane	13.41	10.15	2-(2-hydroxy-2-phenylethoxy)phenol	5.27	5.252
	(Z)-docos-13-enoic acid	42.61	3.36	(3aS,5aR,9aS,9bS)-5a-methyl-3,9-dimethylidene-3a,4,5,6,7,8,9a,9b-octahydrobenzo[g][1]benzofuran-2-one	32.83	4.87	octa-3,5-diyne	5.14	4.29
	2-(butan-2-yldisulfanyl)butane	14.49	2.52	Methyl 9,10-octadecadienoate	37.51	3.01	(Z)-octadec-9-enoic acid	36.81	3.08
	1-(methyldisulfanyl)-1-methylsulfanylpropane	20.77	2.49	methyl 8-[2-[[2-[(2-ethylcyclopropyl)methyl]cyclopropyl]methyl]cyclopropyl]octanoate	40.62	2.72	1-methylsulfanyl-1-(propyldisulfanyl)propane	20.61	3.00
	3-(prop-2-enyltrisulfanyl)prop-1-ene	17.03	2.33	octadec-17-ynoic acid	38.01	1.71	2-(aminomethyl)cyclopentan-1-amine	4.52	2.93
	2-(propyldisulfanyl)butane	13.12	2.21	1-O-dodecyl 10-O-[(2-methylphenyl)methyl] decanedioate	37.68	1.66	2-(propyldisulfanyl)butane	13.15	2.70

**Table:** 

Plant	Essential oil			Methanol extract		
	IUPAC name	RT	% Composition	IUPAC name	RT	% Composition
*Justicia adhatoda*	(E)-octadec-13-enoic acid	36.89	50.76	(Z)-octadec-9-enoic acid	36.83	33.33
	1,3-dihydroxypropan-2-yl octadecanoate	40.68	14.57	oxiran-2-ylmethyl (Z)-octadec-9-enoate	40.67	32.59
	trimethylsilyl (Z)-octadec-9-enoate	37.59	8.92	(3S,8S,9S,10R,13R,14S,17R)-17-[(2R,5S)-5-ethyl-6-methylheptan-2-yl]-10,13-dimethyl-2,3,4,7,8,9, 11,12, 14,15,16,17-dodecahydro-1H-cyclopenta[a]phenanthren-3-ol	42.27	3.94
	(E,7R,11R)-3,7,11,15-tetramethylhexadec-2-en-1-ol	36.24	4.67	Oxiran-2-ylmethyl hexadecanoate	38.58	6.98
	Hexadecanoic acid	34.33	4.22	Docosyl heptanoate	42.57	2.73
	Squalene	44.38	3.72	2-[(Z)-octadec-9-enoxy]ethanol	45.43	1.92
	7,11,15-trimethyl-3-methylidenehexadec-1-ene	32.04	1.98	2,7,8-trimethyl-2-(4,8,12-trimethyltridecyl)-3,4-dihydrochromen-6-ol	48.32	1.56
	Linoelaidic acid	38.03	1.85	(3S,8S,9S,10R,13R,14S,17R)-17-[(E,2R,5S)-5-ethyl-6-methylhept-3-en-2-yl]-10,13-dimethyl-2,3,4,7,8,9,11,12,14,15,16,17-dodecahydro-1H-cyclopenta[a]phenanthren-3-ol (Stigmasterol)	39.01	1.49
	Erucic acid	40.98	1.69	(1R,3aR,5aR,5bR,7aR,9S,11aR,11bR,13aR,13bR)-3a,5a,5b,8,8,11a-hexamethyl-1-prop-1-en-2-yl-1,2,3,4,5,6,7,7a,9,10,11,11b,12,13,13a,13b-hexadecahydrocyclopenta[a]chrysen-9-ol (Lupeol)	45.16	1.43
	Glycidyl palmitate	38.58	1.25			
*Macrotyloma uniflorum*	(Z)-octadec-9-enoic acid	36.87	33.34	-	-	-
	1,3-dihydroxypropan-2-yl octadecanoate	40.71	26.37	-	-	-
	6áBicyclo[4.3.0]nonane, 5á-iodomethyl-1á- isopropenyl-4à,5à-dimethyl-,	45.09	6.85	-	-	-
	2-(3,8-dimethyl-1,2,3,5,6,7,8,8a-octahydroazulen-5-yl)-2-methyloxirane	44.03	3.84	-	-	-
	oxiran-2-ylmethyl hexadecanoate	41.01	4.92	-	-	-
	2-[(Z)-octadec-9-enoxy]ethanol	34.57	2.85	-	-	-
	Hexadecanoic acid	34.36	2.83	-	-	-
	(2R)-2,8-dimethyl-2-[(4R,8R)-4,8,12-trimethyltridecyl]-3,4-dihydrochromen-6-ol	46.50	2.47	-	-	-
	methyl (E)-16-trimethylsilyloxyoctadec-9-enoate	42.63	2.30	-	-	-
	2,7,8-trimethyl-2-(4,8,12-trimethyltridecyl)-3,4-dihydrochromen-6-ol	48.41	2.11	-	-	-
*Nyctanthes arbor-tristis*	5-methyl-1-oxido-2,1,3-benzoxadiazol-1-ium	13.55	9.98	-	-	-
	3-(3-hydroxybutyl)-2,4,4-trimethylcyclohex-2-en-1-one	28.64	9.01	-	-	-
	(2E)-2-benzylideneoctanal	30.63	7.78	-	-	-
	2-methyl-5-prop-1-en-2-ylcyclohexan-1-ol	11.57	6.29	-	-	-
	1-ethyl-8,9-dimethoxy-1,10b-dimethyl-5,6-dihydro-[1,3]oxazolo[4,3-a]isoquinolin-3-one	38.64	5.97	-	-	-
	1-Ethynyl-3,cis(1,1-dimethylethyl)-4,trans-methoxycyclohexan-1-ol	16.95	5.15	-	-	-
	(2E,4E)-hepta-2,4-dien-6-yn-1-ol	10.01	4.27	-	-	-
	3-methyl-2-[(Z)-pent-2-enyl]cyclopent-2-en-1-one	19.79	3.65	-	-	-
	(6E)-3,7,11-trimethyldodeca-1,6,10-trien-3-ol	26.15	3.61	-	-	-
	phenylmethanol	9.97	3.59	-	-	-

## 4 Discussion

The choice of solvent plays a crucial role in determining phytoextraction efficiency, as variations in polarity affect its ability to dissolve specific compounds. The results for phytoextract yield in the present study revealed a clear trend: polar solvents, specifically water and methanol, showed higher recovery than nonpolar solvents. Polar solvents, such as water and methanol, typically yield greater amounts of phenolic compounds and antioxidants than nonpolar solvents like chloroform, due to their ability to extract a diverse array of compounds, including nonphenolic sugars and proteins as well (Hadi et al., 2024). This also indicates a predominance of polar phytoconstituents in these plants (Barchan et al., 2014; Tourabi et al., 2023). The nonpolar solvents consistently showed lower yields across all plants, indicating a smaller quantity of lipophilic compounds or reduced extractability of these fractions. Additionally, solvent selection shapes the profile of extracted phytochemicals, thereby affecting the overall biological activity of the extracts. Plant-based compounds, such as EOs and their bioactive constituents, have been identified as potential biological control agents for reducing and/or controlling phytopathogenic fungi. In this study, we investigated the effects of various plant phytoextracts and EOs against *C. gloeosporioides*, the causative agent of mango anthracnose disease. Strong dose-dependent antifungal activity was observed in most EOs and phytoextracts from different plants, as evidenced by growth inhibition and MIC values. The antifungal bioassay revealed clear dose-dependent trends in spore count reduction across all plant extracts and solvents. AG consistently caused near-complete inhibition at lower concentrations across all solvent extracts, particularly for EO, ME, and some PE treatments, indicating strong fungistatic activity comparable to that of neem and synthetic fungicide azoxystrobin. This robust efficacy highlights its rich profile of volatile and phenolic constituents, as well as strong antifungal potential. FA also performed strongly; however, the anomalies (negative inhibition in *Ferula–*EA, MeOH, TCM) may indicate fungal utilization of solvent-extracted compounds as nutrients at low concentrations, a phenomenon reported in other bioassays (Razzaghi-Abyaneh et al., 2009).

The inhibitory effects of phytoextracts and EOs on the hyphal ultra-morphology were evident in the form of distorted, shriveled, withered, loose, and thin mycelia that were collapsed in the treated groups, whereas the control group had smooth, flat, and homogeneous hyphae. The results indicate that the destruction and collapse of the mycelial walls were due to the effects of various phytoextracts and EOs treatments. Similar distortions in mycelium morphology have previously been reported in several studies (Wang et al., 2005, 2023; Achar et al., 2020). The ultrastructural modifications, particularly the increase in the mycelial distortions, withering, and collapse observed in the present study, are analogous to those produced by synthetic fungicides and other plant extracts (Baka and Mousa, 2020; Anusha et al., 2016; Zobir et al., 2021). In the present study, AG consistently demonstrated the most potent antifungal activity across all tested plant species and extraction solvents. This superior efficacy is supported by the findings showing that AG-EO significantly disrupts fungal cell membranes, induces mitochondrial dysfunction, and elevates reactive oxygen species levels, ultimately leading to cell death (Tian et al., 2012; Wu et al., 2023). FA ranked second in antifungal performance, corroborating previous studies which have been shown to inhibit the growth of multiple fungal pathogens, including *Aspergillus niger*, highlighting its potent antifungal activities owing to the presence of sulfur containing compounds, primarily disulphides, disulfide-thioether, trisulphides as reported in the present study, agree with the previous studies (Divya et al., 2014; Sangchooli et al., 2024). JA consistently occupied a middle rank across treatments, indicating moderate antifungal efficacy. These results can be attributed to its diverse secondary metabolites, including fatty acids, triterpenes, alkaloids, phenolics, flavonoids, terpenoid alcohols, and tocopherols, all of which contribute to antifungal activity (Chernapalli et al., 2024; Hajji-Hedfi et al., 2024). Conversely, MU, NA, and VN consistently clustered in the lower efficacy tier, with VN invariably exhibiting the weakest antifungal effects. Occasional solvent-specific variations were observed, such as NA aligning with MU under aqueous extracts and EOs; however, these fluctuations did not significantly alter the overall efficacy across treatments. Statistical analysis further validated these patterns. Both AG and FA demonstrated highly significant antifungal superiority across all solvent types (*p* < 0.0001), whereas JA showed moderately significant efficacy (*p* < 0.002). Conversely, differences among MU, NA, and VN were statistically non-significant.

A significant solvent effect was evident in this study. Nonpolar solvents (EO, PE) consistently yielded higher inhibition than polar ones (AQ, MeOH, TCM). This suggests that lipophilic antifungal phytochemicals such as terpenes, phenylpropanoids, phenolics, alkaloids, and sesquiterpenes are more efficiently extracted in nonpolar media, aligning with prior findings that EO-rich fractions concentrate bioactive metabolites with strong antifungal activity (Hassan et al., 2021). Conversely, polar extracts may preferentially solubilize glycosides, flavonoids, or polysaccharides that exhibit limited fungitoxicity, explaining the reduced inhibition observed. EOs are rich in monoterpenes, phenolics, and flavonoids, and sesquiterpenes, which disrupt fungal cell membranes, cause oxidative stress, inhibit spore germ tube elongation, and inhibit spore germination (Bakkali et al., 2008; Barchan et al., 2014). Aqueous and methanol extracts capture more polar compounds (sugars, glycosides, tannins), which have weaker or slower antifungal action compared to lipophilic volatiles. These findings highlight a biochemical basis for solvent performance. Nonpolar solvents are more effective at solubilizing hydrophobic bioactive secondary metabolites such as terpenoids, coumarins, and alkaloids, which are well documented for their antifungal properties (Yenjit et al., 2010; Lestari et al., 2024). Monoterpenes and sesquiterpenes, commonly enriched in EO-rich fractions, interfere with fungal cell wall integrity and disrupt membrane permeability, leading to spore lysis and growth inhibition (Maurya et al., 2021). In this study, the consistently higher activity of PE and ethyl ether extracts across species aligns with this chemical principle, suggesting that lipophilic antifungal constituents are central to the observed bioactivity.

Importantly, while solvent effects were significant, dose-dependent inhibition was universal and far more influential than solvent selection, emphasizing that both concentration and extraction medium jointly shape antifungal efficacy. This pattern reinforces earlier reports that concentration-dependent delivery of phytochemicals is critical to overcoming fungal defenses (Khare et al., 2021). Collectively, these results suggest that nonpolar solvent extracts are more effective carriers of potent antifungal compounds, and their use may be strategically important for developing botanical antifungal formulations. However, species-specific differences indicate that phytochemical diversity also modulates solvent efficacy, warranting targeted profiling of individual metabolites in future research. The plant bioactive compounds identified through GC-MS belonged to diverse chemical classes ranging from saturated and unsaturated fatty acids and their esters, terpenes, monoterpenes, triterpenes, sesquiterpenes, phenols, aromatic hydrocarbons, steroidal alcohols, aromatic aldehydes, and ketones, aromatic amines, aromatic esters, macrocyclic lactones, alkenes, diamines, di/trisulphides, and coumarins. The identified compounds were classified into major chemical classes based on their structural features and functional groups. GC–MS profiling of the most potent EOs and phytoextracts revealed a diverse suite of bioactive compounds—primarily unsaturated fatty acids, terpenes, aromatic derivatives, and steroidal alcohols—accounting for 84.6%−97.7% of total active constituents. Notably, the FA-PE extract contained 28 bioactives, whereas NA-EO had 27, emphasizing their rich chemical diversity. EOs with diverse chemical classes (alkaloids, flavonoids, aldehydes, terpenoids, glycosides, ketones, tannins, steroids, fatty acids, carbohydrates, and phenolic compounds) and in different proportions have previously been reported, corroborating our findings (Abubacker and Devi, 2014; He et al., 2018; Kalupahana et al., 2020; Hassan et al., 2021; Silva et al., 2021; Kulkarni et al., 2022; Bhat et al., 2024).

Prominent among these were unsaturated fatty acids such as (Z)-octadec-9-enoic acid (oleic acid), present at ~36.8% in FA-EO and AQ extract, and ~33.3% in MU-EO. Oleic acid and similar unsaturated fatty acids are well-known to disrupt fungal cell membranes, increasing permeability and cellular leakage, thereby inhibiting fungal growth. Awan et al. (2023) reported the antifungal effects of octadecanoic acid against tomato early blight disease caused by *Alternaria solani*. (E)-Octadec-13-enoic acid, comprising ~50.8% in JA-EO and ~33.3% in its MeOH extract, likely exerts comparable effects through membrane destabilization (Yuan et al., 2024). Similar to the present results Purkait et al. (2020) reported the antifungal effects of 13-octadecenoic acid against anthracnose pathogens *Colletotrichum musae* and *C. capsici*. Among aromatic components, AG-EA extract contained 4,7-dimethoxy-5-prop-1-enyl-1,3-benzodioxole (30.1%), and its EO contained 1-ethyl-3-methoxybenzene (~18.6%). Aromatic benzenoids like these have oxidative stress–inducing and enzyme-inhibiting effects that can suppress fungal growth. Eugenol, for example, inhibits the virulence of *C. gloeosporioides* by targeting the laccase enzyme Cglac4, which is critical for pathogenicity (Tan et al., 2025). Tricyclo[4.4.0.0^2^^,^8]dec-3-en-5-ol (~23.1% in AG-EO) is a bicyclic alcohol whose rigid structure may interact with fungal membranes or cell wall components, though specific antifungal data remain limited and require further investigation.

In NA-EO, major constituents such as 5-methyl-1-oxido-2,1,3-benzoxadiazolium (9.98%), 3-(3-hydroxybutyl)-2,4,4-trimethylcyclohex-2-en-1-one (9.01%), and (2E)-2-benzylideneoctanal (7.78%) may disrupt fungal metabolism or signaling via aldehyde- or nitrogen–oxide–mediated protein modifications (Dan et al., 2019). Additional antifungal agents detected in minor quantities include monoterpenes and phenols (α-pinene, limonene, eugenol), which compromise fungal membranes and inhibit spore germination, and triterpenes/steroids (lupeol, stigmasterol), which may interfere with fungal sterol components (Dan et al., 2019; Cheng et al., 2022). The antifungal effects of bioactive compounds from EOs like stigmasterol (Yenjit et al., 2010; Lestari et al., 2024), β-thujene (Treyvaud Amiguet et al., 2006), vaccenic acid (Abdul Salam et al., 2022; Fatima et al., 2023), and limonene (Giménez-Santamarina et al., 2022; de Oliveira Moraes et al., 2025) have been reported.

These multifaceted bioactive profiles align with reports that EOs-rich in terpenes, aromatic constituents, and volatile aldehydes exhibit potent antifungal activity against *C. gloeosporioides* via membrane disruption, ROS generation, enzyme inhibition, and structural damage (Dan et al., 2019; Yuan et al., 2024). Together, these classes suggest a multifaceted mode of action: membrane disruption (fatty acids, terpenoids, epoxides), oxidative and enzymatic inhibition (sulfur compounds, alkaloids), and metabolic interference (steroids). This composite chemical profile likely underpins a synergistic antifungal effect by targeting diverse vulnerability points in the fungal pathogen (Ye et al., 2020). These EO-derived bioactive compounds are regarded as comparatively safer alternatives to synthetic chemical control agents owing to their favorable environmental characteristics, including soil adsorption coefficient (KOC), bioconcentration factor (BCF), metabolic biotransformation half-life in fish (KmHL), and relatively short biodegradation half-life (HL). For instance, environmental fate parameters reported in the COMPTOX database of the United States Environmental Protection Agency for compounds such as (Z)-octadec-11-enoic acid (K_OC_ = 2.34 × 10^3^ L/Kg; BCF = 54.7 L/Kg; KmHL = 0.912 days, and HL = 5.75 days), Hexadecanoic acid (K_OC_ = 1.78 × 10^3^ L/Kg; BCF = 23.9–155 l/Kg); oxiran-2-ylmethyl (Z)-octadec-9-enoate (K_OC_ = 1.35 × 10^4^ L/Kg; BCF = 80–490; KmHL = 2.34 days, and HL = 5.75 days), Squalene (K_OC_ = 5.26 × 10^3^ L/Kg; BCF = 46.1 L/Kg; KmHL = 3.16 days, and HL = 20.9 days), Euricic acid (K_OC_ = 1.38 × 10^4^ L/Kg; BCF = 145 L/Kg; KmHL = 0.38 days, and HL = 5.75 days), Cinnamyl cinnamate (K_OC_ = 1.70x10^3^ L/Kg; BCF = 562 L/Kg; KmHL = 1.7 days, and HL = 3.72 days), further support their comparatively low environmental persistence and safer ecological profile. Furthermore, the beneficial effects of hexadecanoic acid, including mitigation of N_2_O emissions and enhancement of crop productivity, have also been documented (Zhang et al., 2024). Despite their promising safety attributes, EO applications should be approached with caution because the long-term environmental fate and ecological interactions of complex EO mixtures remain insufficiently understood and require extensive investigation. Overall, this study highlights the dual significance of plant-derived metabolites: mechanistic disruption of fungal pathogens and practical potential in sustainable postharvest disease management.

## 5 Conclusions

This study conclusively demonstrated that the choice of plant species and extraction solvent is a critical determinant of both phytoextract yield and antifungal efficacy against *C. gloeosporioides*. A clear dichotomy was observed, with polar solvents (water, methanol) yielding higher extract yields, indicating a predominance of polar phytoconstituents in the studied plants. However, nonpolar solvents and EOs consistently produced extracts with superior antifungal activity. This underscores that efficacy is not a function of yield but of the specific bioactive profile solubilized, with lipophilic compounds–terpenes, sesquiterpenes, aromatic alcohols, ketones, aldehydes, and fatty acids and their derivatives–being primarily responsible for the observed fungitoxicity. Among the plant species evaluated, AG emerged as the most potent antifungal agent, demonstrating significant, dose-dependent inhibition comparable to a synthetic fungicide control. Its efficacy, followed by that of FA, is attributed to a rich profile of volatile and aromatic compounds capable of disrupting fungal cell membranes and inducing oxidative stress, as confirmed by the severe ultrastructural deformities observed in treated hyphae. The antifungal activity was linked to the disruption of mycelial integrity, leading to withering and collapse. GC-MS analysis corroborated these findings, revealing a diverse suite of bioactive compounds, including oleic acid, various terpenoids, and aromatic derivatives, which likely act synergistically to target multiple fungal metabolic and cellular pathways. Moreover, the antifungal potential of these compounds reported in the present study is complemented by their favorable environmental safety profiles, highlighting their suitability for anthracnose control with comparatively lower risks to nontarget organisms. Future research directions should focus on several key areas. First, the development of optimized extraction protocols using nonpolar solvents or sequential extraction methods to maximize the concentration of lipophilic antifungal metabolites. Second, in-depth mechanistic studies are needed to elucidate the specific molecular targets of the most potent identified compounds, such as tricyclo[4.4.0.0^2^^,^8]dec-3-en-5-ol and various disulfides. Third, investigating the synergistic interactions among phytochemicals in the extracts could reveal enhanced efficacy and reduce the required effective dose. Overall, the present findings highlight the potential of the evaluated botanicals for sustainable management of mango anthracnose under controlled experimental conditions. Nevertheless, as *in vivo* validation on mango fruits was not performed in this study, caution is required when extrapolating these results to commercial postharvest systems. Therefore, additional investigations focusing on *in vivo* efficacy, formulation development, storage stability, and large-scale applicability are essential prior to practical implementation. Among the tested treatments, plant-derived EOs from AG and FA exhibited considerable promise as environmentally compatible alternatives to synthetic fungicides and deserve further exploration in sustainable postharvest disease management.

## Data Availability

The original contributions presented in the study are included in the article/supplementary material, further inquiries can be directed to the corresponding authors.
